# Linguistic q-rung orthopair fuzzy group decision-making approach based on new bidirectional projection and generalized knowledge measure

**DOI:** 10.1038/s41598-026-47396-8

**Published:** 2026-05-11

**Authors:** Ya Qin, Yi Liu, Yuan Rong

**Affiliations:** 1 Key Laboratory of Numerical Simulation of Sichuan Provincial Universities, Neijiang Normal University, Neijiang, 641000 Sichuan China; 2 School of Mathematics and Big Data, Neijiang Normal University, Neijiang, 641000 Sichuan China; 3 School of Medical Information and Engineering, Ningxia Medical University, Yinchuan, 750004 Ningxia China

**Keywords:** Linguistic q-ROF set, MCGDM, Normal bidirectional projection, Generalized knowledge measures, Engineering, Mathematics and computing

## Abstract

In response to the challenges of handling linguistic uncertainty in multi-criteria group decision-making (MCGDM), this paper introduces a novel decision framework based on linguistic q-rung orthopair fuzzy (Lq-ROF) sets. The motivations from the need to systematically address ambiguity and inconsistency in linguistic evaluations provided by decision-makers. To this end, the study develops three main methodological contributions. Firstly, a normalized bidirectional projection measure (NBDP) and its weighted extension (WNBDP) are proposed to resolve ranking inconsistencies in linguistic environments. Secondly, an axiomatized knowledge entropy measure for Lq-ROF information is established, enabling fine-grained differentiation among linguistic assessments and facilitating dynamic expert weighting. Thirdly, a non-linear programming model is formulated to objectively derive both attribute and expert weights by integrating bidirectional projection with generalized entropy principles. The proposed framework is rigorously evaluated through comparative studies against established methods, including aggregation-based techniques, Technique for Order Preference by Similarity to Ideal Solution (TOPSIS), Evaluation based on Distance from Average Solution (EDAS), and Complex Proportional Assessment (COPRAS). Results validate the robustness and theoretical advantages of the approach, confirming its effectiveness in quantifying linguistic uncertainty and delivering consistent decision support in complex MCGDM contexts.

## Introduction

China’s shale gas reserves represent a critical component of its national energy strategy, yet their development introduces significant ecological challenges including atmospheric emissions, land degradation, and hydrological impacts. Within the policy framework emphasizing ecological conservation and carbon neutrality, the selection of contractors based on green development capabilities emerges as a strategic decision problem. Formally, a “green contractor” is defined as an agent that employs environmentally sustainable technologies, maximizes resource efficiency, and minimizes ecological disruption throughout project execution. To operationalize this concept, Gu^1^ developed a structured evaluation system consisting of six criteria and multiple sub-indicators, thereby formulating the contractor selection as a MCGDM problem. Globally, the depletion of conventional energy resources coupled with environmental imperatives has elevated shale gas development to a strategic priority. While shale gas offers comparative advantages in energy security and emission profiles relative to conventional fossil fuels, its development entails substantial capital commitment, complex environmental externalizes, and significant socio-economic consequences. These characteristics necessitate rigorous investment risk assessment and project selection mechanisms. The inherent uncertainties, multiple stake holders, and conflicting criteria intrinsic to these evaluations naturally constitute a fuzzy MCGDM problem, requiring the integration of expert judgments under imperfect information^2–7^.

**Table 1:** Green development capability evaluation index system for shale gas development contractors^1^.

Evaluation Index	Secondary indicators
Environmental Protection Measures Capability (C1)	Discharge and treatment of wastewater, exhaust gas, and solid waste (C11)
	Protection of Land and Water Resources (C12)
	Noise Control (C13)
	Water-saving Techniques and Methods (C21)
	Land-saving Techniques and Methods (C22)
Green measures capability (C2)	Material-saving Techniques and Methods (C23)
	Energy-saving Techniques and Methods (C24)
	Production Equipment Maintenance (C25)
	Ground Infrastructure Maintenance (C26)
	Green Development Equipment (C31)
	Green Development Technology (C32)
Advantages of green resources (C3)	Green Development Experience (C33)
	Green Human Resource Reserves (C34)
	Use of Green Materials (C35)
	Corporate Green Strategy Planning (C41)
	Employee Structure and Green Development Training (C42)
Enterprise Management Capability (C4)	On-site Safety Management (C43)
	Integration of Corporate Green Culture (C44)
	HSE (Health, Safety, Environment) Capability (C45)
	Communication and Coordination with Project Stakeholders (C51)
Public Relations and Coordination Capability (C5)	Government Relations (C52)
	Public Relations with Society (C53)
	Corporate Reputation (C54)
	Environmental Investment and Green Technology Investment (C61)
Financial Capability (C6)	Financial Stability (C62)
	Procurement Capability (C63)

Existing research provides valuable methodologies. Hashemizadeh et al.^8^ developed a framework using fuzzy Analytical Network Process (ANP), COPRAS, Multi Attributive Border Approximation Area Comparison (MABAC), and Grey Relational Analysis (GRA) for ranking energy sources under uncertainty. Wu et al.^9^ proposed a hesitant fuzzy linguistic cloud model addressing information vagueness and randomness in project risk assessment. Karamoozian et al.^10^ introduced a hybrid Failure Mode and Effects Analysis (FMEA) approach, while Li et al.^11^ combined system dynamics with fuzzy Analytic Hierarchy Process (AHP). Karamoozian et al.^12^ further contributed a DEMATEL-QUALIFLEX model using interval type-2 hesitant fuzzy sets. These frameworks offer decision support for green contractors of shale gas.

However, a key limitation persists: existing models often overlook investor psychological behavior and lack robust, integrated criteria weighting mechanisms within uncertain environments. This gap is particularly pronounced for shale gas investments, characterized by unique technical, environmental, and regulatory complexities. Consequently, this study aims to bridge these shortcomings by designing a tailored fuzzy MCGDM framework specifically for shale gas investment risk evaluation.

The conflict and uncertainty of actual decision problems pose certain challenges and constraints for decision-makers when analyzing actual decision-making problems. Based on this inspiration, the founding of fuzzy set(FS) theory^13^ effectively characterized the uncertainty in practical problems and laid a theoretical foundation for the study of fuzzy decision. Afterwards, to characterize the ambiguity in practical problems from multiple perspectives and improve the accuracy of decision information, Atanassov^14^ proposed intuitionistic fuzzy set (IFS) theory by extending traditional fuzzy sets to more finely express fuzziness. Although IFS models the vagueness from three aspects of membership, non membership, and hesitancy simultaneously, it also have certain limitations, as the sum of membership and non membership is limited to less than or equal to one. To conquer the above-mentioned shortcomings, the extensions of IFS including inter-valued IFS^15^, Pythagorean fuzzy set (PFS)^16^ including inter-valued PFS^17^ and q-rung orthopair fuzzy (q-ROF) set^18^ are propounded to expand the information expression range and provide more options for experts to provide their preferences. From these extensions, the q-ROF set possesses powerful flexibility and and universal to depict uncertain information and has been investigated to achieve numerous valuable outcomes. In terms of fundamental theory of q-ROF set, the aggregation operators^19,20^, similarity measure^21^, entropy measures^25^ and distance measures^22–24^, decision methodology^26^, and applications of q-ROF set^27–29^ has been studies sequentially.

The introduction of Lq-ROF set^30^ has been thoroughly researched, with its core advantage lying in the introduction of the parameter *q*, which establishes an information description framework with unprecedented expressive power and flexibility for handling uncertainty. This framework is preferentially chosen for many complex decision-making scenarios due to its unique mathematical structure (satisfying the condition $\mu ^q + \nu ^q \le 1$), which significantly expands the information capacity space, enabling precise characterization and computation of highly contradictory or indecisive extreme judgments in human thinking–capabilities that traditional IFS (where $q=1$) and even PFS (where $q=2$) cannot handle. By dynamically adjusting the value of *q*, this framework not only seamlessly integrates with and generalizes the aforementioned various fuzzy set theories but, more importantly, naturally combines qualitative linguistic evaluations (such as “extremely good” but “extremely high risk”) with rigorous quantitative computation. Consequently, in practical high-risk, high-uncertainty applications such as investment evaluation and medical diagnosis, it achieves more authentic and reliable information retention and reasoning, ultimately providing unprecedented modeling capabilities for addressing extremely complex real-world decision-making problems^31–40^.

The investigation of knowledge measures for various fuzzy set models has garnered significant attention in recent research^41,42^. While entropy quantifies the fuzziness of an IFS, Nguyen^43,44^suggested that knowledge measures might function as its dual counterpart; Szmidt et al.^45^. However, subsequent studies by Szmidt and Kacprzyk^45^challenged this notion, proposing instead that a knowledge measure should incorporate both entropy and an uncertainty index. Their framework established that even with a fixed uncertainty index, entropy and knowledge measures do not exhibit a strict dual relationship.

Further developments by Guo^41^ emphasized the distinct nature of entropy and knowledge measures, advocating for their independent treatment through an axiomatic approach. Notably, the axioms introduced in these works exhibit a strong correlation with specific entropy formulations. Despite these advancements, existing knowledge measures for IFSs remain limited in scope-capable of distinguishing only between crisp sets and IFSs but failing to differentiate fuzzy sets from IFSs. This shortcoming arises because the uncertainty index for fuzzy sets is zero, rendering such measures ineffective in such cases. A prevailing consensus in the literature asserts that knowledge measures should monotonically decrease with rising entropy or uncertainty, reflecting an inverse relationship. However, despite this widely accepted principle, no generalized formulation of knowledge measures as a function of both entropy and uncertainty index has yet been established. Ali^46^ The distribution of these uncertainties plays a crucial role in quantifying the knowledge inherent in IFSs.

To address MCDM problems, classical models such as the weighted sum model (WSM), TOPSIS, and VlseKriterijumska Optimizacija I Kompromisno Resenje (VIKOR) have been widely applied across various domains. Given the effectiveness of q-ROF sets in handling uncertainty, researchers have extended these traditional methods to the q-ROF environment, leading to broad practical applications. For instance, Krishankumar et al.^47^ introduced a q-ROF-based COPRAS framework for renewable energy source assessment, while Darko and Liang^48^developed an extended EDAS method using Hamacher operators for group decision-making under q-ROF conditions. Rani and Mishra^49^ proposed a Weighted Aggregated Sum Product ASsessment (WASPAS) approach incorporating a novel score function and similarity measure to evaluate fuel technologies, and Ali^46^ combined Measurement Alternatives and Ranking according to Compromise Solution (MARCOS) and Criteria Importance Through Inter-Criteria correlation (CRITIC) methods for solid waste management. However, these approaches presume fully rational decision-makers, overlooking psychological factors. To account for expert behavior, Gomes^50^ introduced the TODIM method, grounded in prospect theory, to model cognitive biases in decision-making. Subsequently, TODIM has been adapted to various uncertain environments, including Pythagorean fuzzy PFS^51^, hesitant fuzzy set^52^ and others.

Shale gas has gained strategic significance in the global energy transition, yet its development is accompanied by considerable environmental repercussions, such as air pollution, land degradation, and water resource depletion. Against the backdrop of “dual carbon” goals, the selection of contractors with strong green development capabilities has become imperative for achieving sustainable shale gas exploitation.

The motivations of this study can be outlined as follows:

1. Existing Lq-ROF set-based decision methods, particularly those relying on conventional aggregation and projection models, often lead to counterintuitive ranking outcomes and are prone to normalization-related distortions.
2. There remains a lack of integrated modeling capable of synthesizing multiple expert judgments and capturing criterion interdependencies, especially within linguistic uncertainty environments.
3. Current approaches tend to overlook the influence of psychological factors, which play a critical role in attaining reasonable decision outcomes.

To address these research gaps, this paper develops a novel MCGDM framework that integrates bidirectional projection and generalized knowledge measures for the evaluation of green contractors under Lq-ROF environments. The principal contributions of this work are summarized as follows:

1. A normalized bidirectional projection measure is proposed to overcome ranking inconsistencies prevalent in existing methods. Rigorously validated across multiple linguistic scale functions, this measure guarantees intuitive decision outcomes while satisfying essential normalization constraints.
2. A Lq-ROF knowledge entropy measure is established with a axiomatic foundation, enabling discrimination among linguistic evaluations and supporting the dynamic determination of expert weights.
3. A non-linear programming model is constructed to systematically derive attribute weights using the normalized bidirectional projection measure, while expert weights are objectively determined via generalized knowledge-based entropy, ensuring a coherent integration of both weighting processes.
4. A case study on shale gas green contractor selection is presented, demonstrating the operational feasibility and practical applicability of the proposed hybrid decision-making framework in complex energy governance contexts.

The paper is organized into six sections. Section “Introduction” introduces foundational theories of Lq-ROF sets and linguistic scale functions. In Section “Preliminaries”, new normalized bidirectional projection measures and knowledge measures are introduced, along with a comparative analysis of their advantages. Section "Normal bidirectional projection measures and knowledge measures" formulates a non-linear optimization model to calculate attribute weights via normalized bidirectional projection and expert weights through generalized knowledge measures, integrating these into a decision-making algorithm. Section "Decision algorithm under Lq-ROF environment" validates the algorithms practicality through a case study on shale gas green contractor selection, supplemented by comparative and sensitivity analyses. The study concludes in Section “Conclusions” with insights, limitations, and suggestions for future research.

## Preliminaries

This section revisits essential definitions of linguistic scale functions, q-ROFSs, and their linguistic extensions, forming the theoretical foundation for subsequent methodology.

### Linguistic term set and linguistic scale function

In MCDM problems, linguistic term sets (LTS) and linguistic scale functions (LSF) play pivotal roles in quantifying and standardizing the subjective evaluations provided by decision experts (DEs).

An LTS is a discrete, fully ordered set denoted as $S = \{s_0, s_1, \dots , s_t, \dots , s_{2t}\}$, where $2t+1$ represents its granularity. Each term $s_i$ corresponds to a predefined linguistic label (e.g., “poor,” “average”), and *t* is a positive integer. Notably, LTS terms may exhibit semantic overlap, allowing DEs to assign multiple labels for nuanced attribute evaluations. For instance, teaching quality assessments could employ $S = \{\text {extremely poor}, \text {poor}, \text {average}, \text {good}, \text {very good}\}$.

To assign practical meanings to LTS in applications, these terms are convertible into numerical equivalents through the LSF. The purpose of the LSF is to define the semantic value of linguistic terms, making it easier for DEs to express their cognitive information using linguistic terms. The LSF is a mapping function that converts linguistic terms into numerical values, usually represented as $f: S_i \rightarrow \theta _i$. The LSF can be a monotonically increasing function that maintains the order of terms and can be designed differently according to various application scenarios.

In practical applications, LTS and LSF enable DEs to evaluate alternative solutions in a way that is more in line with human cognitive habits. Through the LSF, the linguistic evaluations of deciison makers(DEs) can be transformed into numerical values for subsequent decision analysis and comparison. For instance, if a DE’s assessment of a teacher’s instruction is “good” (with a 30% degree of confidence) and “very good” (with a 50% degree of confidence), these evaluations can be converted into numerical values through the LSF.

The LSF can take various forms, such as those based on subscript functions or prospect theory, each with its own characteristics.

1 $$\begin{aligned}&f_1(s_i)=\frac{i}{2t} (i=0, 1, \cdots , 2t) \end{aligned}$$

2 $$\begin{aligned}&f_2(s_i) = {\left\{ \begin{array}{ll} \frac{a^t-a^{t-i}}{2(a^t-1)} & i=0, 1, \cdots , t; \\ \frac{a^t+a^{i-t}-2}{2(a^t-1)} & i=t+1, t+2, \cdots , 2t. \end{array}\right. } \end{aligned}$$

3 $$\begin{aligned}&f_3(s_i) = {\left\{ \begin{array}{ll} \frac{t^{\alpha }-(t-i)^{\alpha }}{2t^{\alpha }} & i=0, 1, \cdots , t; \\ \frac{t^{\beta }+(i-t)^{\beta }}{2t^{\beta }} & i=t+1, t+2, \cdots , 2t. \end{array}\right. } \end{aligned}$$

Regarding the LSF $f_2$, empirical studies have established that the semantic divergence of consecutive terms grows progressively when moving outward from the central term of an LTS, with parameter *a* typically set to 1.4.

For function $f_3(s_i)$, when $\alpha = \beta = 1$, it reduces to $f_1(s_i)$. This function exhibits an inverse characteristic: the semantic difference between neighboring terms diminishes symmetrically as one moves away from the LTS center.

4 $$\begin{aligned} f_4(s_i) = {\left\{ \begin{array}{ll} \frac{1}{2}e^{(i-t)\alpha } & i=0, 1, \cdots , t; \\ 1-\frac{1}{2}e^{(t-i)\beta } & i=t+1, t+2, \cdots , 2t. \end{array}\right. } \end{aligned}$$

where $\alpha$ represents the metric for one’s inclination towards risks that could lead to gains, whereas $\beta$ signifies the measure for one’s propensity to take risks that might result in losses. The equation $\alpha =\beta$ suggests that the individuals involved in evaluation maintain an identical stance on risk when it comes to judging outcomes labeled as ’positive’ or ’negative’.

In order to preserve the integrity of the original data, Xu^53^ expanded the discrete LTS *S* into a continuous format, denoted as $S=\{s_i|i\in [0, 2t]\}$, which is associated with a continuous LSF *f* that maps $s_i$ to $\theta _i$, with $s_i\in S$. This approach has been similarly adopted by Liu and others. The mentioned LSF function *f* can be represented by $\overline{f}$.

### q-ROFSs and Lq-ROFSs

Orthopair fuzzy sets extend classical fuzzy theory by representing element affiliation through dual membership/non-membership pairs. Yager^18^ introduced the concept of q-ROFS expands this framework through a parameterized constraint space, q-ROFS defined as follows:

#### Definition 1

^18^ For universe *X*, a q-ROFS *Q* is characterized by:

5 $$\begin{aligned} Q = \{(x, u(x), v(x)) \mid x \in X\} \end{aligned}$$

with $u(x),v(x) \in [0,1]$ satisfying $u(x)^q + v(x)^q \le 1$ ($q \ge 1$). The hesitation margin is given by $\pi (x) = (1 - u(x)^q - v(x)^q)^{1/q}$.

Building on q-ROFS and intuitionistic fuzzy sets, the Lq-ROF sets^19^ enables qualitative assessments through predefined LTSs.

#### Definition 2

^19^ For linguistic term set $S = \{s_0,...,s_{2t}\}$, an Lq-ROF set is defined by:

6 $$\begin{aligned} \mathscr {L} = \{(x, s_\alpha (x), s_\beta (x)) \mid x \in X, s_\alpha (x),s_\beta (x) \in S\} \end{aligned}$$

where $\alpha ,\beta$ satisfy $0 \le \alpha ^q + \beta ^q \le (2t)^q$. The linguistic hesitation is $s_\pi$ with $\pi = ((2t)^q - \alpha ^q - \beta ^q)^{1/q}$.

For convenience, the Lq-ROF set $\mathscr {L}_q$ on $X=\{x_1, x_2, \cdots , x_n\}$ denoted as $\mathscr {L}_q=\{(s_{\alpha _i}, s_{\beta _i}, s_{\pi _i})\mid i=1, 2, \cdots , n\}$ with condition $0\le \alpha _i^q + \beta _i^q \le (2t)^q$ and $\pi _i = \root q \of {(2t)^q - \alpha _i^q - \beta _i^q}$. ${{\L } }_q=(s_{\alpha _i}, s_{\beta _i}, s_{\pi _i})$ is called a Lq-ROF number.

In order to compare two Lq-ROF numbers, the score function, accuracy function for Lq-ROF numbers are defined as follows:

#### Definition 3

^19^ For a Lq-ROF number ${{\L } }_q= (s_{\alpha }, s_{\beta })$. The score function $\mathbb {S}({{\L } }_q)$ and accuracy function $\mathbb {A}({{\L } }_q)$ are given by the following Equations:

$$\begin{aligned} \mathbb {S}({{\L } }_q)= & \alpha ^{q}-\beta ^{q}, \\ \mathbb {A}({{\L } }_q)= & \alpha ^{q}-\beta ^{q}. \end{aligned}$$

#### Theorem 1

^19^ Let $({{\L } }_q)_1=(s_{\alpha _1}, s_{\beta _1})$ and $({{\L } }_q)_2=(s_{\alpha _1}, s_{\beta _2})$ be any two Lq-ROF numbers, then the comparison of these two Lq-ROF numbers is defined as follows:

1. If $$\mathbb {S}(({{\L } }_q)_{1}) < \mathbb {S}(({{\L } }_q)_{2})$$, then $$({{\L } }_q)_{1} < ({{\L } }_q)_{2}$$;
2. If $$\mathbb {S}(({{\L } }_q)_{1})> \mathbb {S}(({{\L } }_q)_{2})$$, then $$({{\L } }_q)_{1}> ({{\L } }_q)_{2}$$;
3. If $$\mathbb {S}(({{\L } }_q)_{1}) = \mathbb {S}(({{\L } }_q)_{2})$$, then
  if $$\mathbb {A}(({{\L } }_q)_{1}) < \mathbb {A}(({{\L } }_q)_{2})$$, then $$({{\L } }_q)_{1} < ({{\L } }_q)_{2}$$;
  if $$\mathbb {A}(({{\L } }_q)_{1})> \mathbb {A}(({{\L } }_q)_{2})$$, then $$({{\L } }_q)_{1}> ({{\L } }_q)_{2}$$;
  if $$\mathbb {A}(({{\L } }_q)_{1}) = \mathbb {A}(({{\L } }_q)_{2})$$, then $$({{\L } }_q)_{1} \sim ({{\L } }_q)_{2}$$.

## Normal bidirectional projection measures and knowledge measures

This section first introduces the concept of normal projection measures of Lq-ROF numbers, meanwhile, (weighted) normal bidirectional projection measures and (weighted) generalized knowledge measures are introduced in this section along with their properties.

### Normal projection measures of Lq-ROF numbers

In this section, we develop new inner product and projection measures that extend classical vector space concepts to linguistic decision-making frameworks, with term normalization ensuring scale invariance across evaluation criteria.

#### Definition 4

Let $\mathscr {L}_{q_i}=\{(s_{\alpha _{ij}}, s_{\beta _{ij}}, s_{\pi _{ij}})\} (j=1, 2, \cdots , n; i=1, 2)$ be two Lq-ROF sets which are defined on *X* with *n* elements. Then inner product of $\mathscr {L}_{q_i} (i=1, 2)$ is

7 $$\begin{aligned} IP\left( \mathscr {L}_{q_1}, \mathscr {L}_{q_2}\right) =\sum _{j=1}^{n}\left( (\overline{f}(s_{\alpha _{1j}})\overline{f}(s_{\alpha _{2j}}))^q+(\overline{f}(s_{\beta _{1j}})\overline{f}(s_{\beta _{2j}}))^q+(\overline{f}(s_{\pi _{1j}})\overline{f}(s_{\pi _{2j}}))^q\right) , \end{aligned}$$

The modules of ${{\L } }_{q_i}$ is defined as follows:

8 $$\begin{aligned} M\left( \mathscr {L}_{q_i}\right) = \sqrt{\sum _{j=1}^{n}\left( (\overline{f}(s_{\alpha _{ij}}))^{2q}+(\overline{f}(s_{\beta _{ij}}))^{2q}+(\overline{f}(s_{\pi _{1j}}))^{2q}\right) } \end{aligned}$$

The cosine measure between two Lq-ROF sets ${{\L } }_{q_1}$ and ${{\L } }_{q_2}$ is defined below:

9 $$\begin{aligned}&cos\left( \mathscr {L}_{q_1}, \mathscr {L}_{q_2}\right) =\frac{IP\left( \mathscr {L}_{q_1}, \mathscr {L}_{q_2}\right) }{M(\mathscr {L}_{q_1})M(\mathscr {L}_{q_2})}\nonumber \\&=\frac{\sum _{j=1}^{n}\left( (\overline{f}(s_{\alpha _{1j}})\overline{f}(s_{\alpha _{2j}}))^q+(\overline{f}(s_{\beta _{1j}})\overline{f}(s_{\beta _{2j}}))^q+(\overline{f}(s_{\pi _{1j}})\overline{f}(s_{\pi _{2j}}))^q\right) }{\sqrt{\sum _{j=1}^{n}\left( (\overline{f}(s_{\alpha _{1j}}))^{2q}+(\overline{f}(s_{\beta _{1j}}))^{2q}+(\overline{f}(s_{\pi _{1j}}))^{2q}\right) }\sqrt{\sum _{j=1}^{n}\left( (\overline{f}(s_{\alpha _{2j}}))^{2q}+(\overline{f}(s_{\beta _{2j}}))^{2q}+(\overline{f}(s_{\pi _{2j}}))^{2q}\right) }} \end{aligned}$$

where $$M(\mathscr {L}_{q_1})M(\mathscr {L}_{q_2})\ne 0$$.

According to Eq.(9), the properties of consine measure can be obtained as follows:

(1) $$0 < cos(\mathscr {L}_{q_1}, \mathscr {L}_{q_2})\le 1$$; (2) $$cos(\mathscr {L}_{q_1}, \mathscr {L}_{q_2})= cos(\mathscr {L}_{q_1}, \mathscr {L}_{q_2})$$.

#### Example 1

Let two Lq-ROF sets $\mathscr {L}_{q_1}=\{(s_4, s_2), (s_3, s_3)\}$ and $\mathscr {L}_{q_2}=\{(s_3, s_5), (s_6, s_2)\}$ defined the universal $X=\{x_1, x_2\}$ and $S=\{S_i| i=0, 1, \cdots , 8\}$. Taking $q=2$.

1. If the LSF is $f_1$, then $IP\left( \mathscr {L}_{q_1}, \mathscr {L}_{q_2}\right) =0.7393$, $M(\mathscr {L}_{q_1})=1.05$ and $M|\mathscr {L}_{q_2}|=0.92$, therefore, $cos\left( \mathscr {L}_{q_1}, \mathscr {L}_{q_2}\right) =0.7648$.
2. If the LSF is $f_2$, then $IP\left( \mathscr {L}_{q_1}, \mathscr {L}_{q_2}\right) =0.2938$, $M(\mathscr {L}_{q_1})=0.63$ and $M|\mathscr {L}_{q_2}|=0.84$, therefore, $cos\left( \mathscr {L}_{q_1}, \mathscr {L}_{q_2}\right) =0.5583$.
3. If the LSF is $f_3$, then $IP\left( \mathscr {L}_{q_1}, \mathscr {L}_{q_2}\right) =0.7922$, $M(\mathscr {L}_{q_1})=1.079$ and $M|\mathscr {L}_{q_2}|=0.981$, therefore, $cos\left( \mathscr {L}_{q_1}, \mathscr {L}_{q_2}\right) =0.7486$.
4. If the LSF is $f_4$, then $IP\left( \mathscr {L}_{q_1}, \mathscr {L}_{q_2}\right) =1.273$, $M(\mathscr {L}_{q_1})=1.312$ and $M|\mathscr {L}_{q_2}|=0.417$, therefore, $cos\left( \mathscr {L}_{q_1}, \mathscr {L}_{q_2}\right) =0.6848$.

#### Definition 5

Let $\mathscr {L}_{q_i}=\{(s_{\alpha _{ij}}, s_{\beta _{ij}}, s_{\pi _{ij}})\} (j=1, 2, \cdots , n; i=1, 2)$ be two Lq-ROF sets, which are defined on $X=\{x_1, x_2, \cdots , x_n\}$ and $s_{\alpha _{ij}}, s_{\beta _{ij}}, s_{\pi _{ij}}$ are all in LTS *S* with $2t+1$ granularity. The projection between $\mathscr {L}_{q_1}$ and $\mathscr {L}_{q_2}$ can be defined as follows:

10 $$\begin{aligned} Proj_{\mathscr {L}_{q_2}}(\mathscr {L}_{q_1})= & M\left( \mathscr {L}_{q_1}\right) cos\left( \mathscr {L}_{q_1}, \mathscr {L}_{q_2}\right) \nonumber \\= & \frac{IP(\mathscr {L}_{q_1},\mathscr {L}_{q_2})}{M(\mathscr {L}_{q_2})}\nonumber \\= & \frac{\sum _{j=1}^{n}\left( (\overline{f}(s_{\alpha _{1j}})\overline{f}(s_{\alpha _{2j}}))^q+(\overline{f}(s_{\beta _{1j}})\overline{f}(s_{\beta _{2j}}))^q+(\overline{f}(s_{\pi _{1j}})\overline{f}(s_{\pi _{2j}}))^q\right) }{\sqrt{\sum _{j=1}^{n}\left( (\overline{f}(s_{\alpha _{2j}}))^{2q}+(\overline{f}(s_{\beta _{2j}}))^{2q}+(\overline{f}(s_{\pi _{2j}}))^{2q}\right) }} \end{aligned}$$

As far as the projection measure is concerned, the projection reflects the degree of closeness between two Lq-ROF numbers. The larger the $Proj_{\mathscr {L}_{q_2}}(\mathscr {L}_{q_1})$ value, the closer the $\mathscr {L}_{q_1}$ is to $\mathscr {L}_{q_2}$. Besides, A rigorous projection measure should satisfy the following fundamental mathematical axioms:

1. (Non-Negativity.) That is $Proj_{\mathscr {L}_{q_2}}(\mathscr {L}_{q_1})\ge 0$. This axiom ensures the projection value has a meaningful range, where a value of 0 indicates no similarity or overlap.
2. (Monotonicity) If $\mathscr {L}_{q_1}$ is closer to $\mathscr {L}_{q}$ than $\mathscr {L}_{q_2}$, then: $Proj_{\mathscr {L}_{q}}(\mathscr {L}_{q_1})>Proj_{\mathscr {L}_{q}}(\mathscr {L}_{q_2})$. This is the most direct mathematical expression of the measure’s ability to “reflect similarity” - it monotonically increases as two Lq-ROF numbers become more similar.
3. (Reflexivity) $Proj_{\mathscr {L}_{q_1}}(\mathscr {L}_{q_1})=M\left( \mathscr {L}_{q_1}\right) .$ The projection of a vector onto itself equals its own magnitude, establishing the maximum possible similarity value relative to the vector’s own scale. If $Proj_{\mathscr {L}_{q_1}}(\mathscr {L}_{q_1})=1$ and $Proj_{\mathscr {L}_{q_1}}(\mathscr {L}_{q_2})\in [0,1]$, then the projection is normal.

#### Example 2

(1) Let two Lq-ROF sets $$\mathscr {L}_{q_1}=\{(s_1, s_5), (s_6, s_2)\}$$ and $$\mathscr {L}_{q_2}=\{(s_2, s_5), (s_5, s_2)\}$$ defined the universal $$X=\{x_1, x_2\}$$ and $$S=\{s_i| i=0, 1, \cdots , 8\}$$. Taking $$q=2$$. If the LSF is $$f_1$$, then $$Proj_{\mathscr {L}_{q_2}}(\mathscr {L}_{q_1})=0.9586,$$$$Proj_{\mathscr {L}_{q_2}}(\mathscr {L}_{q_2})=0.9207$$, therefore $$Proj_{\mathscr {L}_{q_2}}(\mathscr {L}_{q_1})> Proj_{\mathscr {L}_{q_2}}(\mathscr {L}_{q_2})$$, which is a contradiction. If the LSF is $$f_4$$, then $$Proj_{\mathscr {L}_{q_2}}(\mathscr {L}_{q_1})=1.4553,$$$$Proj_{\mathscr {L}_{q_2}}(\mathscr {L}_{q_2})=1.423$$, so $$Proj_{\mathscr {L}_{q_2}}(\mathscr {L}_{q_1})> Proj_{\mathscr {L}_{q_2}}(\mathscr {L}_{q_2})$$, which is also a contradiction.        

(2) Let two Lq-ROF sets $$\mathscr {L}_{q_1}=\{(s_4, s_4), (s_5, s_2)\}$$ and $$\mathscr {L}_{q_2}=\{(s_3, s_4), (s_5, s_4)\}$$ defined the universal $$X=\{x_1, x_2\}$$ and $$S=\{S_i| i=0, 1, \cdots , 8\}$$. Taking $$q=2$$. If the LSF is $$f_3$$, then $$Proj_{\mathscr {L}_{q_1}}(\mathscr {L}_{q_2})=0.98,$$$$Proj_{\mathscr {L}_{q_1}}(\mathscr {L}_{q_1})=0.9359$$, so $$Proj_{\mathscr {L}_{q_1}}(\mathscr {L}_{q_2})> Proj_{\mathscr {L}_{q_1}}(\mathscr {L}_{q_1})$$, which is a contradiction. If the LSF is $$f_2$$, then $$Proj_{\mathscr {L}_{q_1}}(\mathscr {L}_{q_2})=1.2483,$$$$Proj_{\mathscr {L}_{q_1}}(\mathscr {L}_{q_1})=0.7728$$, so $$Proj_{\mathscr {L}_{q_1}}(\mathscr {L}_{q_1})> Proj_{\mathscr {L}_{q_1}}(\mathscr {L}_{q_1})$$, which is a contradiction.      

From Example 2, it is not difficult to find that there are following two shortcomings regarding the projection of Lq-ROF sets defined in Definition 2.

1. The projection measures of Lq-ROF sets do not satisfy the normalized condition: $Proj_{\mathscr {L}_{q_2}}(\mathscr {L}_{q_1})\in [0, 1]$;
2. Conventionally, projection magnitudes indicate affinity relationships between entities, with self-projections expected to exceed cross-projections. The counterintuitive phenomenon observed in Example 2, here self-projections register lower magnitudes than projections onto distinct entities, contradicts fundamental expectations.

To rectify these dual limitations, we develop two normalized projection metrics for Lq-ROF sets, extending Ji et al.’s normalization framework^46^ through:

#### Definition 6

Let $\mathscr {L}_{q_i}=\{(s_{\alpha _{ij}}, s_{\beta _{ij}}, s_{\pi _{ij}})\} (j=1, 2, \cdots , n; i=1, 2)$ be two Lq-ROF sets, which are defined on $X=\{x_1, x_2, \cdots , x_n\}$ and $s_{\alpha _{ij}}, s_{\beta _{ij}}, s_{\pi _{ij}}$ are all in LTS *S* with $2t+1$ granularity. The normalized projection between $\mathscr {L}_{q_1}$ and $\mathscr {L}_{q_2}$ can be defined as follows:

11 $$\begin{aligned} NProj_{\mathscr {L}_{q_2}}(\mathscr {L}_{q_1})= \frac{IP(\mathscr {L}_{q_1},\mathscr {L}_{q_2})}{IP(\mathscr {L}_{q_1},\mathscr {L}_{q_2})+\left| M(\mathscr {L}_{q_2})^2-IP(\mathscr {L}_{q_1},\mathscr {L}_{q_2})\right| } \end{aligned}$$

and

12 $$\begin{aligned} GNProj_{\mathscr {L}_{q_2}}(\mathscr {L}_{q_1}) = \frac{1+IP(\mathscr {L}_{q_1},\mathscr {L}_{q_2})}{1+M(\mathscr {L}_{q_1})M(\mathscr {L}_{q_2})+|IP(\mathscr {L}_{q_1},\mathscr {L}_{q_2})-M(\mathscr {L}_{q_2})^2|} \end{aligned}$$

where $$IP\left( \mathscr {L}_{q_1}, \mathscr {L}_{q_2}\right) =\sum _{j=1}^{n}\left( (\overline{f}(s_{\alpha _{1j}})\overline{f}(s_{\alpha _{2j}}))^q+(\overline{f}(s_{\beta _{1j}})\overline{f}(s_{\beta _{2j}}))^q+(\overline{f}(s_{\pi _{1j}})\overline{f}(s_{\pi _{2j}}))^q\right)$$ and

$$M\left( \mathscr {L}_{q_2}\right) = \sqrt{\sum _{j=1}^{n}\left( (\overline{f}(s_{\alpha _{2j}}))^{2q}+(\overline{f}(s_{\beta _{2j}}))^{2q}+(\overline{f}(s_{\pi _{2j}}))^{2q}\right) }$$.

It is obvious that the normalized projection measure is more reasonable than projection measure, and $NProj_{\mathscr {L}_{q_2}}(\mathscr {L}_{q_1})$, $GNProj_{\mathscr {L}_{q_2}}(\mathscr {L}_{q_1})\in [0, 1]$. It is easy to obtain $NProj_{\mathscr {L}_{q}}(\mathscr {L}_{q})=1$ and $GNProj_{\mathscr {L}_{q}}(\mathscr {L}_{q})=1$ for any Lq-ROFS $\mathscr {L}_{q}$.

#### Example 3

(1) Let two Lq-ROF sets $\mathscr {L}_{q_1}=\{(s_1, s_5), (s_6, s_2)\}$ and $\mathscr {L}_{q_2}=\{(s_2, s_5), (s_5, s_2)\}$ defined the universal $X=\{x_1, x_2\}$ and $S=\{S_i| i=0, 1, \cdots , 8\}$. Taking $q=2$. If the LSF is $f_1$, then $NProj_{\mathscr {L}_{q_2}}(\mathscr {L}_{q_1})=0.9619, NProj_{\mathscr {L}_{q_2}}(\mathscr {L}_{q_2})=1$, therefore $NProj_{\mathscr {L}_{q_2}}(\mathscr {L}_{q_1})< NProj_{\mathscr {L}_{q_2}}(\mathscr {L}_{q_2})$. If the LSF is $f_4$, then $NProj_{\mathscr {L}_{q_2}}(\mathscr {L}_{q_1})=0.9785, NProj_{\mathscr {L}_{q_2}}(\mathscr {L}_{q_2})=1$, so $NProj_{\mathscr {L}_{q_2}}(\mathscr {L}_{q_1})< NProj_{\mathscr {L}_{q_2}}(\mathscr {L}_{q_2})$.

(2) Let two Lq-ROF sets $\mathscr {L}_{q_1}=\{(s_4, s_4), (s_5, s_2)\}$ and $\mathscr {L}_{q_2}=\{(s_3, s_4), (s_5, s_4)\}$ defined the universal $X=\{x_1, x_2\}$ and $S=\{S_i| i=0, 1, \cdots , 8\}$. Taking $q=2$. If the LSF is $f_3$, then $NProj_{\mathscr {L}_{q_1}}(\mathscr {L}_{q_2})=0.9172, NProj_{\mathscr {L}_{q_1}}(\mathscr {L}_{q_1})=1$, so $NProj_{\mathscr {L}_{q_1}}(\mathscr {L}_{q_2})< NProj_{\mathscr {L}_{q_1}}(\mathscr {L}_{q_1})$. If the LSF is $f_2$, then $NProj_{\mathscr {L}_{q_1}}(\mathscr {L}_{q_2})=0.9647, NProj_{\mathscr {L}_{q_1}}(\mathscr {L}_{q_1})=1$, so $NProj_{\mathscr {L}_{q_1}}(\mathscr {L}_{q_1})> NProj_{\mathscr {L}_{q_1}}(\mathscr {L}_{q_1})$.

From the Example 3, we can see the normalized projection measures satisfy the mathematical properties: P1, P2 and P3. Therefore, normalized projection measures are more reasonable and consistence with human cognitive.

Also we recalculate Example 1 and list as follows:

#### Example 4

(Continue to Example 1)

1. If the LSF is $f_1$, then $Proj_{\mathscr {L}_{q_2}}(\mathscr {L}_{q_1})=0.8004$, $NProj_{\mathscr {L}_{q_2}}(\mathscr {L}_{q_1})=0.8666$, $GNProj_{\mathscr {L}_{q_2}}(\mathscr {L}_{q_1})=0.8360$.
2. If the LSF is $f_2$, then $Proj_{\mathscr {L}_{q_2}}(\mathscr {L}_{q_1})=0.7598$, $NProj_{\mathscr {L}_{q_2}}(\mathscr {L}_{q_1})=0.9863$, $GNProj_{\mathscr {L}_{q_2}}(\mathscr {L}_{q_1})=0.9301$.
3. If the LSF is $f_3$, then $Proj_{\mathscr {L}_{q_2}}(\mathscr {L}_{q_1})=0.8077$, $NProj_{\mathscr {L}_{q_2}}(\mathscr {L}_{q_1})=0.8235$, $GNProj_{\mathscr {L}_{q_2}}(\mathscr {L}_{q_1})=0.8044$.
4. If the LSF is $f_4$, then $NProj_{\mathscr {L}_{q_2}}(\mathscr {L}_{q_1})=0.8985$, $NProj_{\mathscr {L}_{q_2}}(\mathscr {L}_{q_1})=0.6339$, $GNProj_{\mathscr {L}_{q_2}}(\mathscr {L}_{q_1})=0.6325$.

### Normalized bidirectional projection model

In this section, a novel projection model named bidirectional normal projection model of two Lq-ROF set.

#### Definition 7

Let $\mathscr {L}_{q_i}=\{(s_{\alpha _{ij}}, s_{\beta _{ij}}, s_{\pi _{ij}})\} (j=1, 2, \cdots , n; i=1, 2)$ be two Lq-ROF sets, which are defined on $X=\{x_1, x_2, \cdots , x_n\}$ and $s_{\alpha _{ij}}, s_{\beta _{ij}}, s_{\pi _{ij}}$ are all in LTS *S* with $2t+1$ granularity. The normalized bidirectional projection between $\mathscr {L}_{q_1}$ and $\mathscr {L}_{q_2}$ can be defined as follows:

13 $$\begin{aligned} NBP\left( {\mathscr {L}_{q_2}}, \mathscr {L}_{q_1}\right)= & \frac{1}{1+|Proj_{\mathscr {L}_{q_2}}(\mathscr {L}_{q_1})-Proj_{\mathscr {L}_{q_1}}(\mathscr {L}_{q_2})|} \nonumber \\= & \frac{M(\mathscr {L}_{q_1})M(\mathscr {L}_{q_2})}{M(\mathscr {L}_{q_1})M(\mathscr {L}_{q_2})+|M(\mathscr {L}_{q_1})-M(\mathscr {L}_{q_2})|IP(\mathscr {L}_{q_1},\mathscr {L}_{q_2})} \end{aligned}$$

where $$IP\left( \mathscr {L}_{q_1}, \mathscr {L}_{q_2}\right) =\sum _{j=1}^{n}\left( (\overline{f}(s_{\alpha _{1j}})\overline{f}(s_{\alpha _{2j}}))^q+(\overline{f}(s_{\beta _{1j}})\overline{f}(s_{\beta _{2j}}))^q+(\overline{f}(s_{\pi _{1j}})\overline{f}(s_{\pi _{2j}}))^q\right)$$ and

$$M\left( \mathscr {L}_{q_2}\right) = \sqrt{\sum _{j=1}^{n}\left( (\overline{f}(s_{\alpha _{2j}}))^{2q}+(\overline{f}(s_{\beta _{2j}}))^{2q}+(\overline{f}(s_{\pi _{2j}}))^{2q}\right) }$$.

Obviously, $BNP\left( {\mathscr {L}_{q_1}}, \mathscr {L}_{q_2}\right) \in [0, 1]$. Now, we recalculate the Example 1 by Bidirectional projection model and the calculate results are listed as follows:

#### Example 5

(Continue to Example 1)

1. If the LSF is $f_1$, then $NBP\left( {\mathscr {L}_{q_1}}, \mathscr {L}_{q_2}\right) =0.9141$.
2. If the LSF is $f_2$, then $NBP\left( {\mathscr {L}_{q_1}}, \mathscr {L}_{q_2}\right) =0.8991$.
3. If the LSF is $f_3$, then $NBP\left( {\mathscr {L}_{q_1}}, \mathscr {L}_{q_2}\right) =0.9317$.
4. If the LSF is $f_4$, then $NBP\left( {\mathscr {L}_{q_1}}, \mathscr {L}_{q_2}\right) =0.9327$.

Obviously, Eq.(13) satisfies the normalized condition.

#### Definition 8

Let $\mathscr {L}_{q_i}=\{(s_{\alpha _{ij}}, s_{\beta _{ij}}, s_{\pi _{ij}})\} (j=1, 2, \cdots , n; i=1, 2)$ be two Lq-ROF set, which are defined on $X=\{x_1, x_2, \cdots , x_n\}$ and $s_{\alpha _{ij}}, s_{\beta _{ij}}, s_{\pi _{ij}}$ are all in LTS *S* with $2t+1$ granularity. The weighted normalized bidirectional projection and weighted generalized normalized bidirectional projection between $\mathscr {L}_{q_1}$ and $\mathscr {L}_{q_2}$ can be defined as follows:

14 $$\begin{aligned} WNBP\left( {\mathscr {L}_{q_2}}, \mathscr {L}_{q_1}\right)= & \frac{1}{1+|Proj_{\mathscr {L}_{q_2}}(\mathscr {L}_{q_1})-Proj_{\mathscr {L}_{q_1}}(\mathscr {L}_{q_2})|} \nonumber \\= & \frac{M_w(\mathscr {L}_{q_1})M_w(\mathscr {L}_{q_2})}{M_w(\mathscr {L}_{q_1})M_w(\mathscr {L}_{q_2})+|M_w(\mathscr {L}_{q_1})-M_w(\mathscr {L}_{q_2})|IP_w(\mathscr {L}_{q_1},\mathscr {L}_{q_2})} \end{aligned}$$

and

15 $$\begin{aligned} WGNBP\left( {\mathscr {L}_{q_2}}, \mathscr {L}_{q_1}\right)= & \frac{1+M_w(\mathscr {L}_{q_1})M_w(\mathscr {L}_{q_2})}{1+M_w(\mathscr {L}_{q_1})M_w(\mathscr {L}_{q_2})+|M_w(\mathscr {L}_{q_1})-M_w(\mathscr {L}_{q_2})|IP_w(\mathscr {L}_{q_1},\mathscr {L}_{q_2})} \end{aligned}$$

where

$$\begin{aligned} IP_w\left( \mathscr {L}_{q_1}, \mathscr {L}_{q_2}\right) =\sum _{j=1}^{n}w_j\left( (\overline{f}(s_{\alpha _{1j}})\overline{f}(s_{\alpha _{2j}}))^q+(\overline{f}(s_{\beta _{1j}})\overline{f}(s_{\beta _{2j}}))^q+(\overline{f}(s_{\pi _{1j}})\overline{f}(s_{\pi _{2j}}))^q\right) . \end{aligned}$$

and

$$\begin{aligned} M_w\left( \mathscr {L}_{q_i}\right) = \sqrt{\sum _{j=1}^{n}w_j^2\left( (\overline{f}(s_{\alpha _{ij}}))^{2q}+(\overline{f}(s_{\beta _{ij}}))^{2q}+(\overline{f}(s_{\pi _{ij}}))^{2q}\right) } (i=1, 2). \end{aligned}$$

### Impact of different LSFs on projection measures

In this subsection, some analysis on the different LSFs on projection measures based on Examples 1, 4, and 5.

(**Basic Projection Measures (Example 1)**) The calculation results for inner product, magnitude, and cosine similarity in Example 1 reveal substantial variations in absolute values across different Linguistic Scale Functions (LSFs). The linear function $f_1$ yields moderate similarity values, while the increasing divergence function $f_2$ produces the lowest similarity measures, reflecting its expansive semantic characteristics. In contrast, the decreasing divergence function $f_3$ generates relatively high similarity values, and the exponential function $f_4$ provides moderate-to-high results. This spectrum of outcomes demonstrates that the fundamental projection measures are sensitive to the choice of LSF, with absolute values shifting significantly depending on the underlying semantic interpretation of the linguistic terms.

(**Extended Projection Measures (Example 4)**) The analysis of extended projection measures shows distinct behavioral patterns across the four LSFs. While the basic projection values exhibit relatively small variations, the normalized projection measure displays considerable fluctuations, with $f_2$ yielding the highest value and $f_4$ the lowest. Most notably, the generalized normalized projection measure demonstrates extreme sensitivity, particularly under $f_2$ where an exceptionally high value appears, suggesting potential instability with certain LSF types. These findings indicate that the normalization processes introduce additional layers of sensitivity to the semantic mapping characteristics of different LSFs.

(**Bidirectional Projection Measure (Example 5)**) In contrast to other measures, the normalized bidirectional projection exhibits remarkable stability across all four LSFs. The values remain consistently high with a very narrow range between maximum and minimum values, indicating minimal influence from the choice of linguistic scaling function. This consistency suggests that the bidirectional nature of this measure effectively compensates for variations in semantic interpretation, making it particularly robust for decision-making applications where LSF selection might be uncertain or variable.

The comprehensive analysis reveals a clear hierarchy of sensitivity among the projection measures to LSF selection. While basic and normalized projection measures show moderate to high sensitivity, the bidirectional projection measure demonstrates superior stability. This understanding underscores the importance of measure selection based on application requirements: for scenarios demanding consistency across different semantic interpretations, the bidirectional projection measure is strongly recommended, whereas other measures require careful consideration of LSF characteristics and potential sensitivity testing in practical applications.

### Knowledge-based measures

The application of knowledge-based measures (KBMs) in determining weights provides a method for quantifying the importance of each factor in decision-making. By assessing the amount of information and uncertainty contained in each factor, KBMs assist decision-makers in reasonably allocating weights, ensuring that the decision process reflects the actual impact of each factor,thereby strengthening the analytical rigor and practical utility of decision mechanisms.

#### Definition 9

Let ${{\L } }_{q}=\{(s_{\alpha }, s_{\beta }, s_{\pi })\}$ be a Lq-ROF numbers and $s_{\alpha }, s_{\beta }, s_{\pi }$ are linguistic term in LTS *S* with $2t+1$ granularity. The knowledge-based measure of ${{\L } }_q (LKBM_q)$ is defined as follows:

16 $$\begin{aligned} LKBM_q({{\L } }_q)= & \frac{1}{\sqrt{2}}\sqrt{\overline{f}^{2q}(s_{\alpha })+ \overline{f}^{2q}(s_{\beta })+ (1-\overline{f}^{q}(s_{\pi }))^2}. \end{aligned}$$

From the definition of knowledge-based measure of ${{\L } }_q$, analysis reveals that the q-ROF knowledge measure solely captures information embedded within the membership/non-membership dimensions of q-ROFNs, neglecting the indeterminacy aspect emerging from their divergence. Consequently, extending Nguyen’s^43^ framework, we propose a generalized p-norm knowledge-based measure (q-ROFGKM) for q-ROFSs, alongside a novel entropy metric derived from the q-ROFGKM.

#### Definition 10

Let ${{\L } }_{q}=\{(s_{\alpha }, s_{\beta }, s_{\pi })\}$ be a Lq-ROF numbers and $s_{\alpha }, s_{\beta }, s_{\pi }$ are linguistic term in LTS *S* with $2t+1$ granularity. The generalized p-norm knowledge measure (referred to as q-ROFGKE) for ${{\L } }_{q}$ is given by the normalized aggregate of the generalized p-norm distance and the p-norm fluctuation. The formula for calculating the q-ROFGKE is:

17 $$\begin{aligned} q-ROFGKE({{\L } }_{q}) =\frac{1}{\root p \of {2}+1} \left[ \left( \overline{f}^{pq}(s_{\alpha })+ \overline{f}^{pq}(s_{\beta })+ \left( 1-\overline{f}^{q}(s_{\pi })\right) ^p\right) ^{\frac{1}{p}}+\left| \left( \overline{f}^{q}(s_{\alpha })\right) ^p- \left( \overline{f}^{q}(s_{\beta })\right) ^p\right| ^{\frac{1}{p}}\right] . \end{aligned}$$

Here, the parameter *p* is a real number greater than 1, which signifies the degree of the norm. This measure captures the *p*-norm deviation between the membership and non-membership degrees, thereby reflecting the ambiguity and intrinsic fuzziness inherent in Lq-ROF sets. Utilizing this measure allows for a more precise differentiation among various Lq-ROFSs and aids in ascertaining the weight of decision-makers within the framework of MCGDM.

#### Definition 11

Let ${{\L } }_{q}=\{(s_{\alpha }, s_{\beta }, s_{\pi })\}$ be a Lq-ROF numbers and $s_{\alpha }, s_{\beta }, s_{\pi }$ are linguistic term in LTS *S* with $2t+1$ granularity. The generalized p-norm entropy measure for ${{\L } }_{q}$ is given as follows:

18 $$\begin{aligned} GPE({{\L } }_{q}) =1-\frac{1}{\root p \of {2}+1} \left[ \left( \overline{f}^{pq}(s_{\alpha })+ \overline{f}^{pq}(s_{\beta })+\left( \overline{f}^{q}(s_{\alpha })+\overline{f}^{q}(s_{\beta })\right) ^p\right) ^{\frac{1}{p}}+\left| \left( \overline{f}^{q}(s_{\alpha })\right) ^p- \left( \overline{f}^{q}(s_{\beta })\right) ^p\right| ^{\frac{1}{p}}\right] . \end{aligned}$$

According the Eq.(18), the following properties can be obtained easily.

#### Theorem 2

Let ${{\L } }_{q}=\{(s_{\alpha }, s_{\beta }, s_{\pi })\}$ be a Lq-ROF number, then $GPE ({{\L } }_{q})$ satisfies the following properties:

1. $$GPE ({{\L } }_{q})\in [0, 1]$$;
2. $GPE ({{\L } }_{q})=0$ if and only if $f(s_{\alpha })=1$ or $f(s_{\beta })=1$;
3. $GPE ({{\L } }_{q})=1$ if and only if $f(s_{\alpha })=f(s_{\beta })=0$ and $f(s_{\pi })=1$;
4. $$GPE ({{\L } }_{q})= GPE ({{\L } }_{q}^C)$$.

In above-mentioned properties, if the scale function *f* takes $f_1$, then (P2) and (P3) will reduce to

(P2$^{'}$): $GPE ({{\L } }_{q})=0$ if and only if $s_{\alpha }=S_0$ or $s_{\beta }=S_0$;
(P3$^{'}$): $GPE ({{\L } }_{q})=1$ if and only if $s_{\pi }=S_{2t}$.

## Decision algorithm under Lq-ROF environment

This section establishes a new MCGDM framework for Lq-ROF data, leveraging LSFs, Bi-direction projection and knowledge measures. For convenience, the following information is essential in decision-making problems: Alternatives set $\Gamma =\{\Gamma _1, \Gamma _2, \cdots , \Gamma _m\}$, Attributes set $C=\{C_1, C_2, \cdots , C_n\}$ with weight vector $(w_1, w_2, \cdots , w_n)$ and $w_i\in [0,1],\sum _{i=1}^nw_i=1$. Decision makers set $Dm=\{Dm_1, Dm_2, \cdots , Dm_k\}$ with weight vector $(\lambda _1, \lambda _2, \cdots , \lambda _k)$ and $\lambda _i\in [0,1],\sum _{i=1}^k\lambda _i=1$.

### Determination of attributes’ weights

In this section, the attributes’ weight will be determined as follows:

Step 1. Derive the positive and negative ideal solution of alternatives and denoted as follows

$$\begin{aligned} \Gamma ^+=\left\{ \Gamma _1^+, \Gamma _2^+,\cdots , \Gamma _n^+ \right\} ,\ \ \ \Gamma ^-=\left\{ \Gamma _1^-, \Gamma _2^-,\cdots , \Gamma _n^-\right\} \end{aligned}$$

where $$\Gamma _j=(s_{\alpha _{ij}},s_{\beta _{ij}})$$, $$\Gamma _j^+=\left( max_j(s_{\alpha _{ij}}), min_j(s_{\beta _{ij}})\right)$$ and $$\Gamma _j^-=\left( min_j(s_{\alpha _{ij}}), max_j(s_{\beta _{ij}})\right)$$.

Step 2. To resolve attribute weighting, an optimization framework is developed requiring clear discrimination between alternatives’ projection values relative to PIS/NIS. This objective weighting approach maximizes projection deviation metrics, incorporating both standardized projection measures and incomplete weight information scenarios. The resultant multi-objective programming formulation is:

19 $$\begin{aligned} \max \quad&P(\textbf{w}) = \sum _{i \ne j}^n \left| \text {WGNB}_{\Gamma ^+}(\Gamma _i) - \text {WGNB}_{\Gamma ^+}(\Gamma _j) \right| \end{aligned}$$

20 $$\begin{aligned} \min \quad&N(\textbf{w}) = \sum _{i \ne j}^n \left| \text {WGNB}_{\Gamma ^-}(\Gamma _i) - \text {WGNB}_{\Gamma ^-}(\Gamma _j) \right| \nonumber \\ \text {s.t.} \quad&\sum _{j=1}^{n} w_j = 1, w_j \ge 0, \quad j = 1, 2, \dots , n. \end{aligned}$$

Step 3. The nonlinear programming model to obtain the attribute weights is established as below:

21 $$\begin{aligned} max At(w)=\gamma P(w)-(1-\gamma ) N(w)\nonumber \\ s.t. \sum _{j=1}^{n} w_j =1, w_j\ge 0 . \end{aligned}$$

where $\gamma$ is a balance coefficient and $\gamma \in [0, 1].$ The choice of balance coefficient depends on the Dm’s consideration of the importance of approaches to positive ideal solutions and approaches to negative ideal solutions. If the importance of both objectives is considered equally important, then $\gamma =0.5$. The weight vector $(w_1, w_2, \cdots , w_n)$ of the attributes can obtained by solving the above optimization model.

### Determination of decision makers’ weights

Based upon the proposed GPE, the decision makers’ weights under Lq-ROF environment will be determined as below:

Let the *l*th Dm’s decision information be expressed as $R^l=\left( ({{\L } }_{q}^l)_{ij}\right) _{m\times n}=\left( s_{\alpha _{ij}}^l, s_{\beta _{ij}}^l, s_{\pi _{ij}}^l)_{m\times n}\right)$ under linguistic q-ROF environment. The entropy of the *l*th Dm is defined as below:

$$\begin{aligned} E^l=-\frac{1}{ln(m\times n)}\sum _{i=1}^{m}\sum _{j=1}^{n}\left( \frac{GPE(({{\L } }_{q}^l)_{ij})}{\sum _{i=1}^{m}\sum _{j=1}^{n}GPE(({{\L } }_{q}^l)_{ij})}\right) ln\left( \frac{GPE (({{\L } }_{q}^l)_{ij})}{\sum _{i=1}^{m}\sum _{j=1}^{n}GPE(({{\L } }_{q}^l)_{ij})}\right) . \end{aligned}$$

The weight $\lambda _l$ of the lth Dm can be expressed by

22 $$\begin{aligned} \lambda _l=\frac{1-E^l}{1-\sum _{l=1}^{k}E^l}, \end{aligned}$$

where *k* means the total number of Dms.

### Presented decision algorithm

**Fig. 1:**
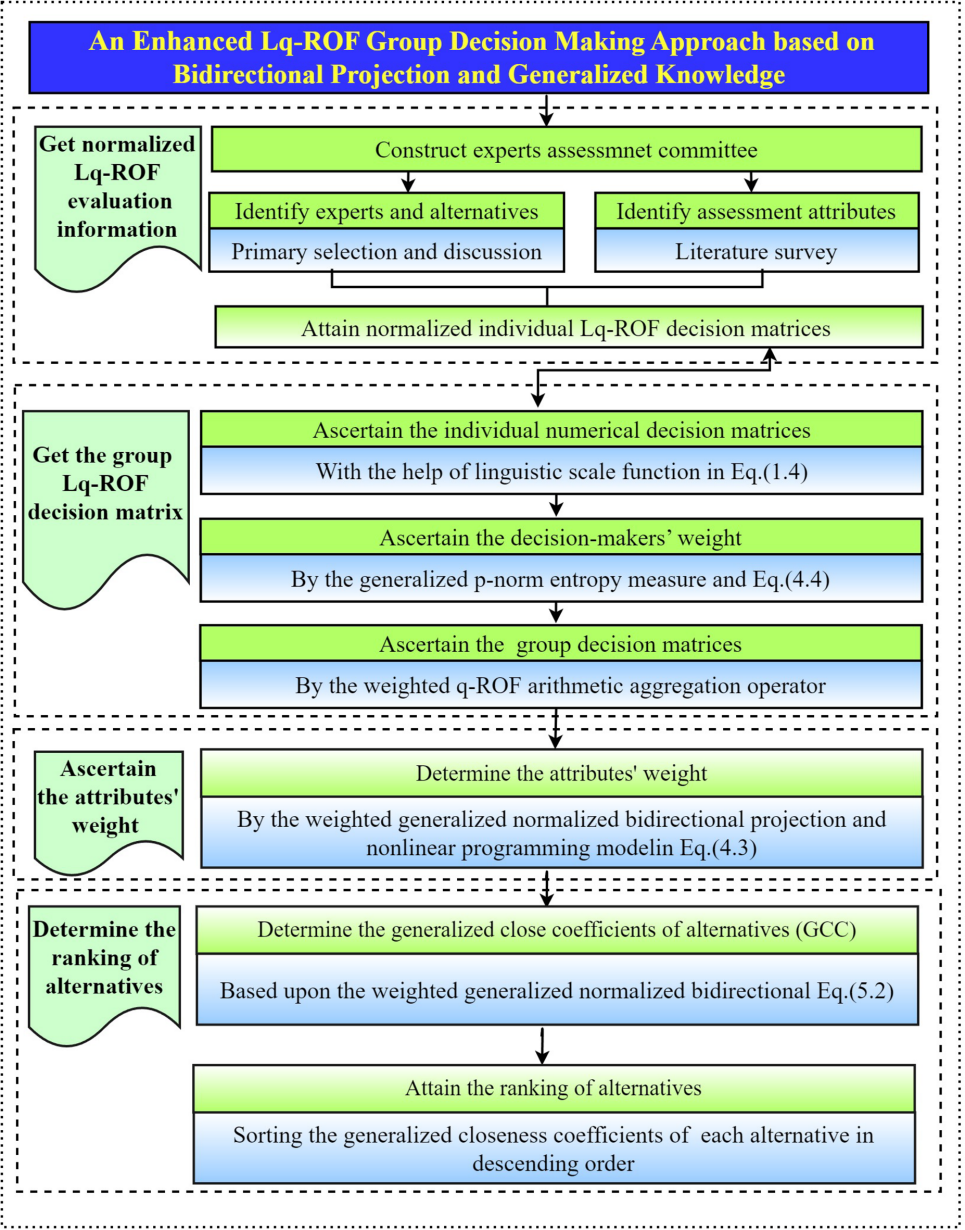
The flowchart of the propounded Linguistic q-ROF decision algorithm.

Now, according to the approaches of weights of attributes and experts in Section "Decision algorithm under Lq-ROF environment", the MCGDM method is improved and the specific implementation process is listed as below. Further, a visual framework diagram of the proposed propounded decision algorithm under Lq-ROF environment is portrayed in Fig. 1.

**Step 1:** Normalized individual decision making matrix $R^k=\left( ({{\L } }_{q}^k)_{ij})\right) _{m\times n}=\left( \left( s_{\alpha _{ij}}^k, s_{\beta _{ij}}^k\right) _{m\times n}\right)$ and obtained $\tilde{R}^k = \left( ({{\L } }_{q}^k)_{ij}\right) _{m\times n}$, where $(\tilde{{\L } }_{q}^k)_{ij} =({{\L } }_{q}^k)_{ij}$ when the *j*th attribute $c_j$ is benefit-type, $(\tilde{{\L } }_{q}^k)_{ij} =\left( ({{\L } }_{q}^k)_{ij}\right) ^c$ when the *j*th attribute $c_j$ is cost-type.

**Step 2:** By linguistic scale functions $f_4$, transform the original decision matrices to numerical decision matrices.

**Step 3:** According to the generalized knowledge measures proposed in Section 4, determine the decision-makers’ weight $(\lambda _1, \lambda _2, \cdots , \lambda _k)$ by Eq. (22).

**Step 4:** Collective group decision matrices is derived from the individual decision matrices for the criteria by employing the following operator:

23 $$\begin{aligned} Lq((\tilde{{\L } }_{q}^1)_{ij}, (\tilde{{\L } }_{q}^2)_{ij}, \cdots , (\tilde{{\L } }_{q}^k)_{ij})=\lambda _1(\tilde{{\L } }_{q}^1)_{ij}\bigoplus \lambda _2(\tilde{{\L } }_{q}^2)_{ij}\bigoplus \cdots \bigoplus \lambda _k(\tilde{{\L } }_{q}^k)_{ij} \end{aligned}$$

**Step 5:** The decision matrices for criterion (Step 4) are aggregated into a collective decision matrix using weighted q-ROF arithmetic aggregation operators.

**Step 6:** Construct nonlinear programming model to address the attributes’ weights.

**Step 7:** Alternatives are ranked by computing generalized close coefficients (GCC) based on WGNBP values;

24 $$\begin{aligned} GCC_i=\frac{WGNBP_{\Gamma ^+}(\Gamma _i)}{WGNBP_{\Gamma ^+}(\Gamma _i)+WGNBP_{\Gamma ^-}(\Gamma _i)}, i=1, 2, 3, 4. \end{aligned}$$

### Illustrative example

This section demonstrates the practical application of the proposed approach through a case study assessing the green development capacity of shale gas contractors’. To further validate the method, comparative analyzes and sensitivity tests are conducted to examine its effectiveness and stability.

A shale gas development company needs to choose a green contractor, and currently there are four contractors $\Gamma _i(i=1,2,3,4$) available. The evaluation system for green capability development for shale gas contractors is shown in Table 1, and shale gas development companies organize relevant experts to evaluate it. Four contractors $\Gamma _i$ ($i=1,2,3,4$) and six decision criteria $C_j (j=1, 2, 3, 4, 5, 6)$ are ascertained. Three decision makers $Dm=\{Dm_1, Dm_2, Dm_3\}$ from different fields are determined, the background, experiences of them are illustrated in Table 2. The secondary impact indicator $I_{jk_j}$ under ($j=1,2,3,4,5,6; k_j=1,2,\cdots , n_j; n_1=3, n_2=6, n_3=n_4=5, n_5=4, n_6=3$). The evaluation information is given by the lq-ROF numbers with granularity of LTS $S=\{s_i| i=0, 1, \cdots , 6\}$ 7. And the results of the evaluation according to each criterion are shown in Tables 3,4,5,6,7,8,9,10,11,12,13,14,15,16,17,18,19,20.

**Table 2:** The basic information of decision makers $Dm=\{Dm_1, Dm_2, Dm_3\}$.

Decision makers	Background	Degree	Experience
$Dm_1$	Industrial engineering	Doctor’s degree	8 years
$Dm_2$	Energy management	Doctor’s degree	8 years
$Dm_3$	Shale gas supply	Master’s degree	10 years

**Table 3:** Individual Lq-ROF decision matrix regarding criteria $C_1$ given by $E_1$

$\Gamma _i$	$C_{11}$	$C_{12}$	$C_{13}$
$\Gamma _1$	($s_5$, $s_1$)	($s_4$, $s_1$)	($s_5$, $s_2$)
$\Gamma _2$	($s_5$, $s_0$)	($s_5$, $s_1$)	($s_5$, $s_1$)
$\Gamma _3$	($s_6$, $s_1$)	($s_6$, $s_2$)	($s_4$, $s_2$)
$\Gamma _4$	($s_5$, $s_2$)	($s_4$, $s_0$)	($s_4$, $s_0$)

**Table 4:** Individual Lq-ROF decision matrix regarding criteria $C_2$ by $E_1$.

$\Gamma _i$	$C_{21}$	$C_{22}$	$C_{23}$	$C_{24}$	$C_{25}$	$C_{26}$
$\Gamma _1$	($s_5$, $s_0$)	($s_4$, $s_1$)	($s_4$, $s_1$)	($s_5$, $s_2$)	($s_4$, $s_2$)	($s_5$, $s_0$)
$\Gamma _2$	($s_6$, $s_2$)	($s_5$, $s_2$)	($s_5$, $s_2$)	($s_4$, $s_0$)	($s_3$, $s_1$)	($s_4$, $s_1$)
$\Gamma _3$	($s_5$, $s_1$)	($s_5$, $s_3$)	($s_4$, $s_0$)	($s_5$, $s_2$)	($s_4$, $s_1$)	($s_4$, $s_2$)
$\Gamma _4$	($s_5$, $s_2$)	($s_3$, $s_1$)	($s_4$, $s_1$)	($s_5$, $s_4$)	($s_6$, $s_3$)	($s_5$, $s_2$)

**Table 5:** Individual Lq-ROF decision matrix regarding criteria $C_3$ by $E_1$.

$\Gamma _i$	$C_{31}$	$C_{32}$	$C_{33}$	$C_{34}$	$C_{35}$
$\Gamma _1$	($s_6$, $s_1$)	($s_4$, $s_1$)	($s_5$, $s_0$)	($s_4$, $s_1$)	($s_5$, $s_0$)
$\Gamma _2$	($s_5$, $s_2$)	($s_5$, $s_1$)	($s_5$, $s_2$)	($s_5$, $s_0$)	($s_4$, $s_1$)
$\Gamma _3$	($s_5$, $s_1$)	($s_5$, $s_2$)	($s_5$, $s_2$)	($s_5$, $s_2$)	($s_4$, $s_0$)
$\Gamma _4$	($s_5$, $s_0$)	($s_4$, $s_0$)	($s_4$, $s_1$)	($s_5$, $s_1$)	($s_3$, $s_0$)

**Table 6:** Individual Lq-ROF decision matrix regarding criteria $C_4$ by $E_1$.

$\Gamma _i$	$C_{41}$	$C_{42}$	$C_{43}$	$C_{44}$	$C_{45}$
$\Gamma _1$	($s_5$, $s_0$)	($s_5$, $s_1$)	($s_4$, $s_0$)	($s_4$, $s_1$)	($s_6$, $s_0$)
$\Gamma _2$	($s_5$, $s_1$)	($s_5$, $s_1$)	($s_5$, $s_1$)	($s_5$, $s_1$)	($s_4$, $s_0$)
$\Gamma _3$	($s_4$, $s_1$)	($s_5$, $s_2$)	($s_5$, $s_1$)	($s_5$, $s_0$)	($s_4$, $s_1$)
$\Gamma _4$	($s_5$, $s_0$)	($s_4$, $s_0$)	($s_4$, $s_0$)	($s_4$, $s_1$)	($s_3$, $s_0$)

**Table 7:** Individual Lq-ROF decision matrix regarding criteria $C_5$ by $E_1$.

$\Gamma _i$	$C_{51}$	$C_{52}$	$C_{53}$	$C_{54}$
$\Gamma _1$	($s_5$, $s_2$)	($s_5$, $s_0$)	($s_4$, $s_1$)	($s_5$, $s_1$)
$\Gamma _2$	($s_4$, $s_1$)	($s_5$, $s_1$)	($s_5$, $s_1$)	($s_4$, $s_1$)
$\Gamma _3$	($s_4$, $s_0$)	($s_4$, $s_1$)	($s_5$, $s_0$)	($s_5$, $s_2$)
$\Gamma _4$	($s_5$, $s_1$)	($s_5$, $s_1$)	($s_5$, $s_0$)	($s_5$, $s_1$)

### Contractor assessment decision process

The *q*-value was maintained at 3 throughout all computational stages of this selection process.

**Table 8:** Individual Lq-ROF decision matrix regarding criteria $C_6$ by $E_1$.

$\Gamma _i$	$C_{61}$	$C_{62}$	$C_{63}$
$\Gamma _1$	($s_5$, $s_2$)	($s_5$, $s_1$)	($s_5$, $s_2$)
$\Gamma _2$	($s_6$, $s_1$)	($s_5$, $s_0$)	($s_4$, $s_1$)
$\Gamma _3$	($s_5$, $s_0$)	($s_4$, $s_1$)	($s_5$, $s_0$)
$\Gamma _4$	($s_5$, $s_1$)	($s_5$, $s_2$)	($s_5$, $s_2$)

**Table 9:** Individual Lq-ROF decision matrix regarding criteria $C_1$ given by $E_2$.

$\Gamma _i$	$C_{11}$	$C_{12}$	$C_{13}$
$\Gamma _1$	($s_5$, $s_0$)	($s_4$, $s_0$)	($s_5$, $s_1$)
$\Gamma _2$	($s_5$, $s_2$)	($s_5$, $s_1$)	($s_5$, $s_2$)
$\Gamma _3$	($s_6$, $s_0$)	($s_5$, $s_1$)	($s_4$, $s_0$)
$\Gamma _4$	($s_5$, $s_1$)	($s_4$, $s_1$)	($s_5$, $s_0$)

**Table 10:** Individual Lq-ROF decision matrix regarding criteria $C_2$ by $E_2$.

$\Gamma _i$	$C_{21}$	$C_{22}$	$C_{23}$	$C_{24}$	$C_{25}$	$C_{26}$
$\Gamma _1$	($s_5$, $s_1$)	($s_4$, $s_1$)	($s_5$, $s_2$)	($s_4$, $s_0$)	($s_5$, $s_0$)	($s_5$, $s_1$)
$\Gamma _2$	($s_6$, $s_0$)	($s_5$, $s_1$)	($s_4$, $s_1$)	($s_4$, $s_0$)	($s_3$, $s_1$)	($s_4$, $s_1$)
$\Gamma _3$	($s_5$, $s_1$)	($s_5$, $s_2$)	($s_4$, $s_0$)	($s_5$, $s_1$)	($s_4$, $s_0$)	($s_4$, $s_0$)
$\Gamma _4$	($s_5$, $s_2$)	($s_4$, $s_1$)	($s_4$, $s_1$)	($s_5$, $s_1$)	($s_5$, $s_0$)	($s_5$, $s_1$)

**Table 11:** Individual Lq-ROF decision matrix regarding criteria $C_3$ by $E_2$.

$\Gamma _i$	$C_{31}$	$C_{32}$	$C_{33}$	$C_{34}$	$C_{35}$
$\Gamma _1$	($s_6$, $s_0$)	($s_5$, $s_0$)	($s_4$, $s_2$)	($s_5$, $s_0$)	($s_5$, $s_0$)
$\Gamma _2$	($s_5$, $s_1$)	($s_5$, $s_1$)	($s_5$, $s_0$)	($s_6$, $s_0$)	($s_5$, $s_1$)
$\Gamma _3$	($s_4$, $s_1$)	($s_5$, $s_0$)	($s_4$, $s_1$)	($s_5$, $s_2$)	($s_4$, $s_1$)
$\Gamma _4$	($s_4$, $s_0$)	($s_4$, $s_1$)	($s_5$, $s_1$)	($s_5$, $s_2$)	($s_5$, $s_2$)

**Table 12:** Individual Lq-ROF decision matrix regarding criteria $C_4$ by $E_2$.

$\Gamma _i$	$C_{41}$	$C_{42}$	$C_{43}$	$C_{44}$	$C_{45}$
$\Gamma _1$	($s_5$, $s_1$)	($s_5$, $s_2$)	($s_4$, $s_1$)	($s_4$, $s_1$)	($s_5$, $s_2$)
$\Gamma _2$	($s_5$, $s_2$)	($s_5$, $s_1$)	($s_5$, $s_1$)	($s_5$, $s_2$)	($s_5$, $s_1$)
$\Gamma _3$	($s_5$, $s_0$)	($s_3$, $s_1$)	($s_4$, $s_1$)	($s_4$, $s_1$)	($s_5$, $s_2$)
$\Gamma _4$	($s_5$, $s_2$)	($s_4$, $s_1$)	($s_5$, $s_0$)	($s_4$, $s_1$)	($s_4$, $s_1$)

**Table 13:** Individual Lq-ROF decision matrix regarding criteria $C_5$ by $E_2$.

$\Gamma _i$	$C_{51}$	$C_{52}$	$C_{53}$	$C_{54}$
$\Gamma _1$	($s_4$, $s_1$)	($s_4$, $s_1$)	($s_5$, $s_2$)	($s_5$, $s_1$)
$\Gamma _2$	($s_5$, $s_1$)	($s_5$, $s_2$)	($s_5$, $s_1$)	($s_5$, $s_0$)
$\Gamma _3$	($s_5$, $s_0$)	($s_5$, $s_0$)	($s_4$, $s_1$)	($s_5$, $s_2$)
$\Gamma _4$	($s_5$, $s_2$)	($s_4$, $s_1$)	($s_4$, $s_0$)	($s_4$, $s_1$)

**Table 14:** Individual Lq-ROF decision matrix regarding criteria $C_6$ by $E_2$.

$\Gamma _i$	$C_{61}$	$C_{62}$	$C_{63}$
$\Gamma _1$	($s_4$, $s_1$)	($s_5$, $s_1$)	($s_6$, $s_0$)
$\Gamma _2$	($s_5$, $s_0$)	($s_4$, $s_1$)	($s_5$, $s_2$)
$\Gamma _3$	($s_5$, $s_1$)	($s_5$, $s_2$)	($s_4$, $s_1$)
$\Gamma _4$	($s_4$, $s_1$)	($s_4$, $s_2$)	($s_4$, $s_0$)

**Step 1:** Normalized individual decision making matrix $R^k=\left( I_{ij}^k\right) _{m\times n}=\left( \left( s_{\alpha _{ij}}^k, s_{\beta _{ij}}^k\right) _{m\times n}\right)$ and obtained $\tilde{R}^k = \left( \tilde{I}_{ij}^k \right) _{m\times n}$, where $k=1,2,3,4$.

**Table 15:** Individual Lq-ROF decision matrix regarding criteria $C_1$ given by $E_3$.

$\Gamma _i$	$C_{11}$	$C_{12}$	$C_{13}$
$\Gamma _1$	($s_5$, $s_2$)	($s_4$, $s_0$)	($s_5$, $s_1$)
$\Gamma _2$	($s_5$, $s_1$)	($s_4$, $s_1$)	($s_5$, $s_2$)
$\Gamma _3$	($s_5$, $s_2$)	($s_4$, $s_1$)	($s_5$, $s_1$)
$\Gamma _4$	($s_5$, $s_1$)	($s_4$, $s_1$)	($s_4$, $s_1$)

**Table 16:** Individual Lq-ROF decision matrix regarding criteria $C_2$ by $E_3$.

$\Gamma _i$	$C_{21}$	$C_{22}$	$C_{23}$	$C_{24}$	$C_{25}$	$C_{26}$
$\Gamma _1$	($s_6$, $s_0$)	($s_4$, $s_1$)	($s_4$, $s_2$)	($s_5$, $s_1$)	($s_4$, $s_1$)	($s_5$, $s_1$)
$\Gamma _2$	($s_5$, $s_2$)	($s_5$, $s_1$)	($s_5$, $s_1$)	($s_4$, $s_1$)	($s_3$, $s_1$)	($s_4$, $s_1$)
$\Gamma _3$	($s_5$, $s_1$)	($s_5$, $s_1$)	($s_4$, $s_0$)	($s_5$, $s_2$)	($s_4$, $s_1$)	($s_4$, $s_2$)
$\Gamma _4$	($s_5$, $s_2$)	($s_3$, $s_1$)	($s_4$, $s_1$)	($s_5$, $s_2$)	($s_5$, $s_3$)	($s_5$, $s_2$)

**Table 17:** Individual Lq-ROF decision matrix regarding criteria $C_3$ by $E_3$.

$\Gamma _i$	$C_{31}$	$C_{32}$	$C_{33}$	$C_{34}$	$C_{35}$
$\Gamma _1$	($s_5$, $s_0$)	($s_6$, $s_1$)	($s_5$, $s_0$)	($s_5$, $s_1$)	($s_5$, $s_2$)
$\Gamma _2$	($s_4$, $s_1$)	($s_5$, $s_2$)	($s_4$, $s_2$)	($s_4$, $s_0$)	($s_4$, $s_1$)
$\Gamma _3$	($s_5$, $s_2$)	($s_4$, $s_1$)	($s_5$, $s_0$)	($s_5$, $s_1$)	($s_5$, $s_0$)
$\Gamma _4$	($s_5$, $s_0$)	($s_4$, $s_1$)	($s_4$, $s_1$)	($s_5$, $s_2$)	($s_4$, $s_2$)

**Table 18:** Individual Lq-ROF decision matrix regarding criteria $C_4$ by $E_3$.

$\Gamma _i$	$C_{41}$	$C_{42}$	$C_{43}$	$C_{44}$	$C_{45}$
$\Gamma _1$	($s_6$, $s_0$)	($s_5$, $s_3$)	($s_5$, $s_0$)	($s_4$, $s_1$)	($s_4$, $s_1$)
$\Gamma _2$	($s_4$, $s_1$)	($s_5$, $s_0$)	($s_5$, $s_1$)	($s_5$, $s_0$)	($s_5$, $s_0$)
$\Gamma _3$	($s_5$, $s_1$)	($s_4$, $s_1$)	($s_4$, $s_1$)	($s_5$, $s_1$)	($s_5$, $s_2$)
$\Gamma _4$	($s_5$, $s_2$)	($s_4$, $s_2$)	($s_5$, $s_2$)	($s_5$, $s_0$)	($s_5$, $s_2$)

**Table 19:** Individual Lq-ROF decision matrix regarding criteria $C_5$ by $E_3$.

$\Gamma _i$	$C_{51}$	$C_{52}$	$C_{53}$	$C_{54}$
$\Gamma _1$	($s_5$, $s_0$)	($s_5$, $s_0$)	($s_6$, $s_1$)	($s_5$, $s_0$)
$\Gamma _2$	($s_4$, $s_1$)	($s_5$, $s_2$)	($s_5$, $s_2$)	($s_4$, $s_1$)
$\Gamma _3$	($s_3$, $s_1$)	($s_4$, $s_1$)	($s_5$, $s_2$)	($s_5$, $s_1$)
$\Gamma _4$	($s_4$, $s_1$)	($s_4$, $s_1$)	($s_4$, $s_1$)	($s_5$, $s_2$)

**Step 2:** By linguistic scale functions $f_4$, transform the original decision matrices to following decision matrices, listed in Tables 21,22,23,24,25,26,27,28,29,30,31,32,33,34,35,36,37,38.

**Table 20:** Individual Lq-ROF decision matrix regarding criteria $C_6$ by $E_3$.

$\Gamma _i$	$C_{61}$	$C_{62}$	$C_{63}$
$\Gamma _1$	($s_6$, $s_1$)	($s_5$, $s_0$)	($s_5$, $s_1$)
$\Gamma _2$	($s_5$, $s_0$)	($s_4$, $s_1$)	($s_4$, $s_1$)
$\Gamma _3$	($s_5$, $s_2$)	($s_4$, $s_1$)	($s_3$, $s_1$)
$\Gamma _4$	($s_4$, $s_2$)	($s_5$, $s_2$)	($s_4$, $s_1$)

**Table 21:** Individual decision matrix LSF-based regarding criteria $C_1$ given by $E_1$.

$\Gamma _i$	$C_{11}$	$C_{12}$	$C_{13}$
$\Gamma _1$	(0.91, 0.09)	(0.79, 0.09)	(0.91, 0.21)
$\Gamma _2$	(0.91, 0.21)	(0.91, 0.09)	(0.91, 0.09)
$\Gamma _3$	(0.91, 0.09)	(0.50, 0.21)	(0.79, 0.21)
$\Gamma _4$	(0.91, 0.21)	(0.79, 0.21)	(0.79, 0.21)

**Table 22:** Individual decision matrix LSF-based regarding criteria $C_2$ by $E_1$.

$\Gamma _i$	$C_{21}$	$C_{22}$	$C_{23}$	$C_{24}$	$C_{25}$	$C_{26}$
$\Gamma _1$	(0.91, 0.21)	(0.50, 0.09)	(0.79, 0.09)	(0.91, 0.21)	(0.79, 0.21)	(0.91, 0.04)
$\Gamma _2$	(0.79, 0.09)	(0.91, 0.21)	(0.79, 0.21)	(0.79, 0.09)	(0.50, 0.09)	(0.50, 0.09)
$\Gamma _3$	(0.79, 0.09)	(0.91, 0.50)	(0.79, 0.21)	(0.91, 0.21)	(0.79, 0.09)	(0.79, 0.21)
$\Gamma _4$	(0.91, 0.21)	(0.50, 0.09)	(0.79, 0.09)	(0.50, 0.50)	(0.50, 0.50)	(0.91, 0.21)

**Table 23:** Individual decision matrix LSF-based regarding criteria $C_3$ by $E_1$.

$\Gamma _i$	$C_{31}$	$C_{32}$	$C_{33}$	$C_{34}$	$C_{35}$
$\Gamma _1$	(0.91, 0.09)	(0.79, 0.09)	(0.91, 0.04)	(0.79, 0.09)	(0.91, 0.04)
$\Gamma _2$	((0.79, 0.21)	(0.79, 0.09)	(0.91, 0.21)	(0.79, 0.21)	(0.79, 0.09)
$\Gamma _3$	(0.91, 0.09)	(0.91, 0.50)	(0.91, 0.50)	(0.91, 0.50)	(0.79, 0.04)
$\Gamma _4$	(0.91, 0.21)	(0.79, 0.21)	(0.79, 0.09)	(0.91, 0.09)	(0.50, 0.09)

**Table 24:** Individual decision matrix LSF-based regarding criteria $C_4$ by $E_1$.

$\Gamma _i$	$C_{41}$	$C_{42}$	$C_{43}$	$C_{44}$	$C_{45}$
$\Gamma _1$	(0.91, 0.21)	(0.91, 0.09)	(0.79, 0.21)	(0.79, 0.09)	(0.79, 0.21)
$\Gamma _2$	(0.91, 0.09)	(0.91, 0.21)	(0.79, 0.09)	(0.91, 0.09)	(0.79, 0.04)
$\Gamma _3$	(0.79, 0.09)	(0.79, 0.21)	(0.79, 0.09)	(0.79, 0.21)	(0.79, 0.21)
$\Gamma _4$	(0.91, 0.21)	(0.79, 0.09)	(0.79, 0.09)	(0.79, 0.09)	(0.50, 0.21)

**Table 25:** Individual decision matrix LSF-based regarding criteria $C_5$ by $E_1$.

$\Gamma _i$	$C_{51}$	$C_{52}$	$C_{53}$	$C_{54}$
$\Gamma _1$	(0.91,0.21)	(0.91,0.09)	(0.91,0.21)	(0.79,0.09)
$\Gamma _2$	(0.91,0.09)	(0.79,0.09)	(0.91,0.09)	(0.79,0.09)
$\Gamma _3$	(0.50,0.04)	(0.79,0.09)	(0.79,0.04)	(0.91,0.09)
$\Gamma _4$	(0.79,0.21)	(0.79,0.21)	(0.91,0.09)	(0.79,0.21)

**Step 3:** According to the generalized knowledge measures proposed in Section "Decision algorithm under Lq-ROF environment", determine decision-makers’ weight by Eq. (22) and listed as below: $\lambda _1=0.346, \lambda _2=0.318, \lambda _3=0.336.$

**Table 26:** Individual decision matrix LSF-based regarding criteria $C_6$ by $E_1$.

$\Gamma _i$	$C_{61}$	$C_{62}$	$C_{63}$
$\Gamma _1$	(0.91,0.21)	(0.91,0.09)	(0.91,0.21)
$\Gamma _2$	(0.79,0.09)	(0.91,0.09)	(0.79,0.09)
$\Gamma _3$	(0.50,0.04)	(0.79,0.09)	(0.79,0.04)
$\Gamma _4$	(0.91,0.09)	(0.79,0.21)	(0.79,0.21)

**Table 27:** Individual decision matrix LSF-based regarding criteria $C_1$ given by $E_2$.

$\Gamma _i$	$C_{11}$	$C_{12}$	$C_{13}$
$\Gamma _1$	(0.91,0.04)	(0.79,0.04)	(0.91,0.09)
$\Gamma _2$	(0.91,0.21)	(0.91,0.09)	(0.91,0.21)
$\Gamma _3$	(0.91,0.50)	(0.91,0.09)	(0.79,0.09)
$\Gamma _4$	(0.91,0.09)	(0.79,0.09)	(0.91,0.21)

**Table 28:** Individual decision matrix LSF-based regarding criteria $C_2$ by $E_2$.

$\Gamma _i$	$C_{21}$	$C_{22}$	$C_{23}$	$C_{24}$	$C_{25}$	$C_{26}$
$\Gamma _1$	(0.91,0.09)	(0.79,0.09)	(0.91,0.21)	(0.79,0.21)	(0.91,0.21)	(0.91,0.09)
$\Gamma _2$	(0.91,0.50)	(0.91,0.09)	(0.79,0.09)	(0.79,0.21)	(0.50,0.09)	(0.79,0.09)
$\Gamma _3$	(0.91,0.09)	(0.91,0.21)	(0.79,0.09)	(0.91,0.09)	(0.79,0.50)	(0.79,0.50)
$\Gamma _4$	(0.91,0.21)	(0.79,0.09)	(0.79,0.09)	(0.91,0.09)	(0.91,0.21)	(0.91,0.09)

**Table 29:** Individual decision matrix LSF-based regarding criteria $C_3$ by $E_2$.

$\Gamma _i$	$C_{31}$	$C_{32}$	$C_{33}$	$C_{34}$	$C_{35}$
$\Gamma _1$	(0.91,0.50)	(0.91,0.50)	(0.79,0.21)	(0.91,0.50)	(0.91,0.50)
$\Gamma _2$	(0.91,0.09)	(0.91,0.09)	(0.91,0.50)	(0.91,0.50)	(0.91,0.09)
$\Gamma _3$	(0.79,0.09)	(0.91,0.50)	(0.79,0.21)	(0.91,0.21)	(0.79,0.21)
$\Gamma _4$	(0.91,0.50)	(0.79,0.09)	(0.91,0.09)	(0.91,0.21)	(0.91,0.21)

**Table 30:** Individual decision matrix LSF-based regarding criteria $C_4$ by $E_2$.

$\Gamma _i$	$C_{41}$	$C_{42}$	$C_{43}$	$C_{44}$	$C_{45}$
$\Gamma _1$	(0.91,0.09)	(0.91,0.21)	(0.79,0.21)	(0.79,0.21)	(0.91,0.21)
$\Gamma _2$	(0.91,0.21)	(0.91,0.21)	(0.91,0.21)	(0.91,0.21)	(0.91,0.21)
$\Gamma _3$	(0.91,0.50)	(0.50,0.09)	(0.79,0.21)	(0.79,0.21)	(0.91,0.21)
$\Gamma _4$	(0.91,0.21)	(0.79,0.09)	(0.91,0.50)	(0.79,0.21)	(0.79,0.09)

**Step 4:** Collective group decision matrices is derived from the individual decision matrices for the criteria by employing Eq. (23) and the collective group matrices are listed in Tables 39,40,41,42,43,44.

**Table 31:** Individual decision matrix LSF-based regarding criteria $C_5$ by $E_2$.

$\Gamma _i$	$C_{51}$	$C_{52}$	$C_{53}$	$C_{54}$
$\Gamma _1$	(0.91,0.09)	(0.91,0.50)	(0.91,0.21)	(0.91,0.09)
$\Gamma _2$	(0.91,0.50)	(0.91,0.21)	(0.91,0.09)	(0.91,0.21)
$\Gamma _3$	(0.91,0.50)	(0.91,0.21)	(0.79,0.09)	(0.91,0.21)
$\Gamma _4$	(0.91,0.21)	(0.79,0.21)	(0.91,0.21)	(0.91,0.50)

**Table 32:** Individual decision matrix LSF-based regarding criteria $C_6$ by $E_2$.

$\Gamma _i$	$C_{61}$	$C_{62}$	$C_{63}$
$\Gamma _1$	(0.79,0.21)	(0.91,0.09)	(0.79,0.21)
$\Gamma _2$	(0.91,0.21)	(0.79,0.50)	(0.91,0.21)
$\Gamma _3$	(0.91,0.21)	(0.91,0.50)	(0.91,0.21)
$\Gamma _4$	(0.91,0.21)	(0.91,0.21)	(0.91,0.09)

**Table 33:** Individual decision matrix LSF-based regarding criteria $C_1$ given by $E_3$.

$\Gamma _i$	$C_{11}$	$C_{12}$	$C_{13}$
$\Gamma _1$	(0.91,0.21)	(0.79,0.04)	(0.91,0.09)
$\Gamma _2$	(0.91,0.09)	(0.79,0.09)	(0.91,0.21)
$\Gamma _3$	(0.91,0.21)	(0.79,0.09)	(0.91,0.09)
$\Gamma _4$	(0.91,0.09)	(0.79,0.09)	(0.79,0.09)

**Table 34:** Individual decision matrix LSF-based regarding criteria $C_2$ by $E_3$.

$\Gamma _i$	$C_{21}$	$C_{22}$	$C_{23}$	$C_{24}$	$C_{25}$	$C_{26}$
$\Gamma _1$	(0.96,0.04)	(0.79,0.09)	(0.79,0.21)	(0.91,0.09)	(0.79,0.09)	(0.91,0.09)
$\Gamma _2$	(0.91,0.21)	(0.91,0.09)	(0.91,0.09)	(0.79,0.09)	(0.50,0.09)	(0.79,0.09)
$\Gamma _3$	(0.91,0.09)	(0.91,0.09)	(0.79,0.04)	(0.91,0.21)	(0.79,0.09)	(0.79,0.21)
$\Gamma _4$	(0.91,0.21)	(0.50,0.09)	(0.79,0.09)	(0.91,0.21)	(0.91,0.50)	(0.91,0.21)

**Table 35:** Individual decision matrix LSF-based regarding criteria $C_3$ by $E_3$.

$\Gamma _i$	$C_{31}$	$C_{32}$	$C_{33}$	$C_{34}$	$C_{35}$
$\Gamma _1$	(0.91,0.04)	(0.79,0.09)	(0.91,0.04)	(0.91,0.09)	(0.91,0.21)
$\Gamma _2$	(0.79,0.09)	(0.91,0.21)	(0.79,0.21)	(0.79,0.04)	(0.79,0.09)
$\Gamma _3$	(0.91,0.21)	(0.79,0.09)	(0.91,0.04)	(0.91,0.09)	(0.91,0.04)
$\Gamma _4$	(0.91,0.04)	(0.79,0.09)	(0.79,0.09)	(0.91,0.21)	(0.79,0.21)

**Step 5:** Aggregating the collective group decision matrices regarding criteria $c_j (j=1, 2, \cdots , 6)$ by applying weighted q-ROF arithmetic aggregation operators under the condition that the weights are equal for each secondary indicators, the results are listed in Table 45 and determine the PIS and NIS matrix, the results is listed in Table 46.

**Table 36:** Individual decision matrix LSF-based regarding criteria $C_4$ by $E_3$.

$\Gamma _i$	$C_{41}$	$C_{42}$	$C_{43}$	$C_{44}$	$C_{45}$
$\Gamma _1$	(0.96,0.04)	(0.91,0.50)	(0.91,0.04)	(0.79,0.09)	(0.79,0.09)
$\Gamma _2$	(0.79,0.09)	(0.91,0.04)	(0.91,0.09)	(0.91,0.04)	(0.91,0.04)
$\Gamma _3$	(0.91,0.09)	(0.79,0.09)	(0.79,0.09)	(0.91,0.09)	(0.91,0.21)
$\Gamma _4$	(0.91,0.21)	(0.79,0.21)	(0.91,0.21)	(0.91,0.04)	(0.91,0.21)

**Table 37:** Individual decision matrix LSF-based regarding criteria $C_5$ by $E_3$.

$\Gamma _i$	$C_{51}$	$C_{52}$	$C_{53}$	$C_{54}$
$\Gamma _1$	(0.91,0.04)	(0.91,0.04)	(0.79,0.09)	(0.91,0.04)
$\Gamma _2$	(0.79,0.09)	(0.91,0.21)	(0.91,0.21)	(0.79,0.09)
$\Gamma _3$	(0.50,0.09)	(0.79,0.09)	(0.91,0.21)	(0.91,0.09)
$\Gamma _4$	(0.79,0.09)	(0.79,0.09)	(0.79,0.09)	(0.91,0.21)

**Table 38:** Individual decision matrix LSF-based regarding criteria $C_6$ by $E_3$.

$\Gamma _i$	$C_{61}$	$C_{62}$	$C_{63}$
$\Gamma _1$	(0.79,0.09)	(0.91,0.04)	(0.91,0.09)
$\Gamma _2$	(0.91,0.04)	(0.79,0.09)	(0.79,0.09)
$\Gamma _3$	(0.91,0.21)	(0.79,0.09)	(0.50,0.09)
$\Gamma _4$	(0.79,0.21)	(0.91,0.21)	(0.79,0.09)

**Table 39:** Lq-ROF decision matrix regarding criteria $C_1$.

$\Gamma _i$	$C_{11}$	$C_{12}$	$C_{13}$
$\Gamma _1$	(0.91,0.09)	(0.79,0.05)	(0.91,0.12)
$\Gamma _2$	(0.91,0.16)	(0.89,0.09)	(0.91,0.15)
$\Gamma _3$	(0.91,0.20)	(0.80,0.12)	(0.85,0.12)
$\Gamma _4$	(0.91,0.12)	(0.79,0.12)	(0.85,0.16)

**Table 40:** Individual Lq-ROF decision matrix regarding criteria $C_2$.

$\Gamma _i$	$C_{21}$	$C_{22}$	$C_{23}$	$C_{24}$	$C_{25}$	$C_{26}$
$\Gamma _1$	(0.94,0.09)	(0.73,0.09)	(0.85,0.15)	(0.89,0.16)	(0.85,0.16)	(0.91,0.06)
$\Gamma _2$	(0.88,0.20)	(0.91,0.12)	(0.85,0.12)	(0.79,0.11)	(0.50,0.09)	(0.73,0.09)
$\Gamma _3$	(0.88,0.09)	(0.91,0.21)	(0.79,0.09)	(0.91,0.16)	(0.79,0.15)	(0.79,0.27)
$\Gamma _4$	(0.91,0.21)	(0.64,0.09)	(0.79,0.09)	(0.86,0.21)	(0.86,0.38)	(0.91,0.16)

**Step 6:** Construct nonlinear programming model to address the attributes’ weights.

$${\left\{ \begin{array}{ll} \begin{aligned} & \max At(w) = \gamma P(w) - (1 - \gamma ) N(w), \\ & \max P(w) = \sum _{\begin{array}{c} i,j=1 \\ i\ne j \end{array}}^{4} \left| WGNB_{\Gamma ^+}(\Gamma _i) - WGNBP_{\Gamma ^+}(\Gamma _j) \right| , \\ & \min N(w) = \sum _{\begin{array}{c} i,j=1 \\ i\ne j \end{array}}^{4} \left| WGNBP_{\Gamma ^-}(\Gamma _i) - WGNBP_{\Gamma ^-}(\Gamma _j) \right| , \\ & \text {s.t.} \quad \sum _{j=1}^{6} w_j = 1, \quad w_j \ge 0. \end{aligned} \end{array}\right. }$$

in which,

$$\begin{aligned} WGNBP_{\Gamma ^+}(\Gamma _i)=\frac{1+M_w(\Gamma _i)M_w(\Gamma ^+)}{1+M_w(\Gamma _1)M_w(\Gamma ^+)+|M_w(\Gamma _1)-M_w(\Gamma ^+)|IP_w(\Gamma _i, \Gamma ^+)},\\ WGNBP_{\Gamma ^-}(\Gamma _i)=\frac{1+M_w(\Gamma _i)M_w(\Gamma ^-)}{1+M_w(\Gamma _1)M_w(\Gamma ^-)+|M_w(\Gamma _1)-M_w(\Gamma ^-)|IP_w(\Gamma _i, \Gamma ^-)},\\ M_w(\Gamma _1)=\sqrt{0.48w_1^2+0.45w_2^2+0.5w_3^2+0.46w_4^2+0.53w_5^2+0.49w_6^2},\\ M_w(\Gamma _2)=\sqrt{0.55w_1^2+0.31w_2^2+0.41w_3^2+0.52w_4^2+0.45w_5^2+0.41w_6^2},\\ M_w(\Gamma _3)=\sqrt{0.42w_1^2+0.41w_2^2+0.49w_3^2+0.35w_4^2+0.44w_5^2+0.35w_6^2},\\ M_w(\Gamma _4)=\sqrt{0.41w_1^2+0.38w_2^2+0.42w_3^2+0.40w_4^2+0.46w_5^2+0.45w_6^2},\\ M_w(\Gamma ^+)=\sqrt{0.55w_1^2+0.45w_2^2+0.5w_3^2+0.52w_4^2+0.53w_5^2+0.49w_6^2},\\ M_w(\Gamma ^-)=\sqrt{0.41w_1^2+0.31w_2^2+0.41w_3^2+0.35w_4^2+0.44w_5^2+0.35w_6^2},\\ IP_w(\Gamma _1, \Gamma ^+)=0.52w_1+0.45w_2+0.5w_3+0.49w_4+0.53w_5+0.49w_6,\\ IP_w(\Gamma _2, \Gamma ^+)=0.55w_1+0.37w_2+0.46w_3+0.52w_4+0.49w_5+0.45w_6,\\ IP_w(\Gamma _3, \Gamma ^+)=0.48w_1+0.43w_2+0.50w_3+0.43w_4+0.48w_5+0.41w_6,\\ IP_w(\Gamma _4, \Gamma ^+)=0.48w_1+0.42w_2+0.46w_3+0.46w_4+0.49w_5+0.47w_6,\\ IP_w(\Gamma _1, \Gamma ^-)=0.45w_1+0.37w_2+0.46w_3+0.4w_4+0.48w_5+0.41w_6,\\ IP_w(\Gamma _2, \Gamma ^-)=0.48w_1+0.31w_2+0.41w_3+0.43w_4+0.44w_5+0.38w_6,\\ IP_w(\Gamma _3, \Gamma ^-)=0.41w_1+0.36w_2+0.45w_3+0.35w_4+0.44w_5+0.35w_6,\\ IP_w(\Gamma _4, \Gamma ^-)=0.41w_1+0.35w_2+0.42w_3+0.37w_4+0.45w_5+0.39w_6. \end{aligned}$$

the results are obtained as follows (fixed $\gamma =0.4$): $w_1=0.159, w_2= 0.200, w_3= 0.212, w_4= 0.131, w_5= 0.164, w_6= 0.133$

**Step 7:** Ranking the schemes according to the weighted normalized bidirectional projection,

$$\begin{aligned} GCC_i=\frac{WGNBP_{\Gamma ^+}(\Gamma _i)}{WGNBP_{\Gamma ^+}(\Gamma _i)+WGNBP_{\Gamma ^-}(\Gamma _i)}, i=1,2,3,4. \end{aligned}$$

when $\gamma =0.4$, the results are $GCC_1=0.71, GCC_2= 0.67, GCC_3=0.61, GCC_4=0.62.$ Therefore, the order of the schemes is $\Gamma _1> \Gamma _2> \Gamma _4> \Gamma _3.$

**Table 41:** Individual Lq-ROF decision matrix regarding criteria $C_3$.

$\Gamma _i$	$C_{31}$	$C_{32}$	$C_{33}$	$C_{34}$	$C_{35}$
$\Gamma _1$	(0.91,0.11)	(0.85,0.15)	(0.89,0.06)	(0.88,0.15)	(0.91,0.15)
$\Gamma _2$	(0.85,0.12)	(0.88,0.12)	(0.89,0.27)	(0.85,0.15)	(0.85,0.09)
$\Gamma _3$	(0.89,0.12)	(0.89,0.28)	(0.89,0.16)	(0.91,0.21)	(0.85,0.06)
$\Gamma _4$	(0.91,0.15)	(0.79,0.12)	(0.85,0.09)	(0.91,0.15)	(0.80,0.15)

**Table 42:** Individual Lq-ROF decision matrix regarding criteria $C_4$.

$\Gamma _i$	$C_{41}$	$C_{42}$	$C_{43}$	$C_{44}$	$C_{45}$
$\Gamma _1$	(0.94,0.09)	(0.91,0.20)	(0.85,0.12)	(0.79,0.11)	(0.85,0.16)
$\Gamma _2$	(0.89,0.11)	(0.91,0.12)	(0.88,0.11)	(0.91,0.09)	(0.88,0.06)
$\Gamma _3$	(0.88,0.15)	(0.74,0.12)	(0.79,0.11)	(0.85,0.16)	(0.88,0.21)
$\Gamma _4$	(0.91,0.21)	(0.79,0.12)	(0.88,0.20)	(0.85,0.09)	(0.81,0.16)

**Table 43:** Individual Lq-ROF decision matrix regarding criteria $C_5$.

$\Gamma _i$	$C_{51}$	$C_{52}$	$C_{53}$	$C_{54}$
$\Gamma _1$	(0.91,0.09)	(0.91,0.15)	(0.85,0.11)	(0.91,0.06)
$\Gamma _2$	(0.85,0.15)	(0.88,0.15)	(0.91,0.12)	(0.85,0.11)
$\Gamma _3$	(0.81,0.21)	(0.85,0.11)	(0.89,0.21)	(0.91,0.16)
$\Gamma _4$	(0.89,0.11)	(0.79,0.16)	(0.89,0.21)	(0.91,0.27)

**Table 44:** Individual Lq-ROF decision matrix regarding criteria $C_6$.

$\Gamma _i$	$C_{61}$	$C_{62}$	$C_{63}$
$\Gamma _1$	(0.85,0.16)	(0.91,0.06)	(0.89,0.16)
$\Gamma _2$	(0.88,0.09)	(0.85,0.15)	(0.85,0.11)
$\Gamma _3$	(0.86,0.11)	(0.85,0.15)	(0.81,0.08)
$\Gamma _4$	(0.89,0.15)	(0.88,0.21)	(0.85,0.12)

**Table 45:** Collective decision matrix.

$\Gamma _i$	$C_{1}$	$C_{2}$	$C_{3}$	$C_{4}$	$C_{5}$	$C_{6}$
$\Gamma _1$	(0.89,0.08)	(0.88,0.11)	(0.89,0.12)	(0.88,0.13)	(0.90,0.13)	(0.89,0.12)
$\Gamma _2$	(0.91,0.13)	(0.82,0.12)	(0.86,0.14)	(0.90,0.10)	(0.88,0.13)	(0.86,0.11)
$\Gamma _3$	(0.86,0.14)	(0.86,0.15)	(0.89,0.15)	(0.84,0.15)	(0.87,0.17)	(0.84,0.11)
$\Gamma _4$	(0.86,0.13)	(0.85,0.16)	(0.87,0.13)	(0.86,0.15)	(0.88,0.18)	(0.87,0.15)

**Table 46:** PIS and NIS.

$\Gamma _i$	$C_{1}$	$C_{2}$	$C_{3}$	$C_{4}$	$C_{5}$	$C_{6}$
$\Gamma ^+$	(0.91,0.08)	(0.88,0.11)	(0.89,0.12)	(0.90,0.10)	(0.90,0.13)	(0.89,0.11)
$\Gamma ^-$	(0.86,0.14)	(0.82,0.16)	(0.86,0.15)	(0.84,0.15)	(0.87,0.18)	(0.84,0.15)

**Table 47:** Correlation coefficients for Different $\gamma$ Values.

$\gamma$	$GCC_1$	$GCC_2$	$GCC_3$	$GCC_4$	Rangking
0.1	0.63	0.59	0.54	0.56	$\Gamma _1> \Gamma _2> \Gamma _4> \Gamma _3$
0.2	0.64	0.63	0.55	0.57	$\Gamma _1> \Gamma _2> \Gamma _4> \Gamma _3$
0.3	0.69	0.68	0.60	0.59	$\Gamma _1> \Gamma _2> \Gamma _4> \Gamma _3$
0.4	0.71	0.67	0.61	0.62	$\Gamma _1> \Gamma _2> \Gamma _4> \Gamma _3$
0.5	0.73	0.61	0.61	0.68	$\Gamma _1> \Gamma _4> \Gamma _2> \Gamma _3$
0.6	0.69	0.75	0.62	0.65	$\Gamma _2> \Gamma _1> \Gamma _4> \Gamma _3$
0.7	0.71	0.77	0.64	0.66	$\Gamma _2> \Gamma _1> \Gamma _4> \Gamma _3$
0.8	0.72	0.78	0.65	0.68	$\Gamma _2> \Gamma _1> \Gamma _4> \Gamma _3$
0.9	0.73	0.79	0.66	0.69	$\Gamma _2> \Gamma _1> \Gamma _4> \Gamma _3$
1.0	0.74	0.80	0.68	0.70	$\Gamma _2> \Gamma _1> \Gamma _4> \Gamma _3$

#### Parameter sensitivity analysis

**Fig. 2:**
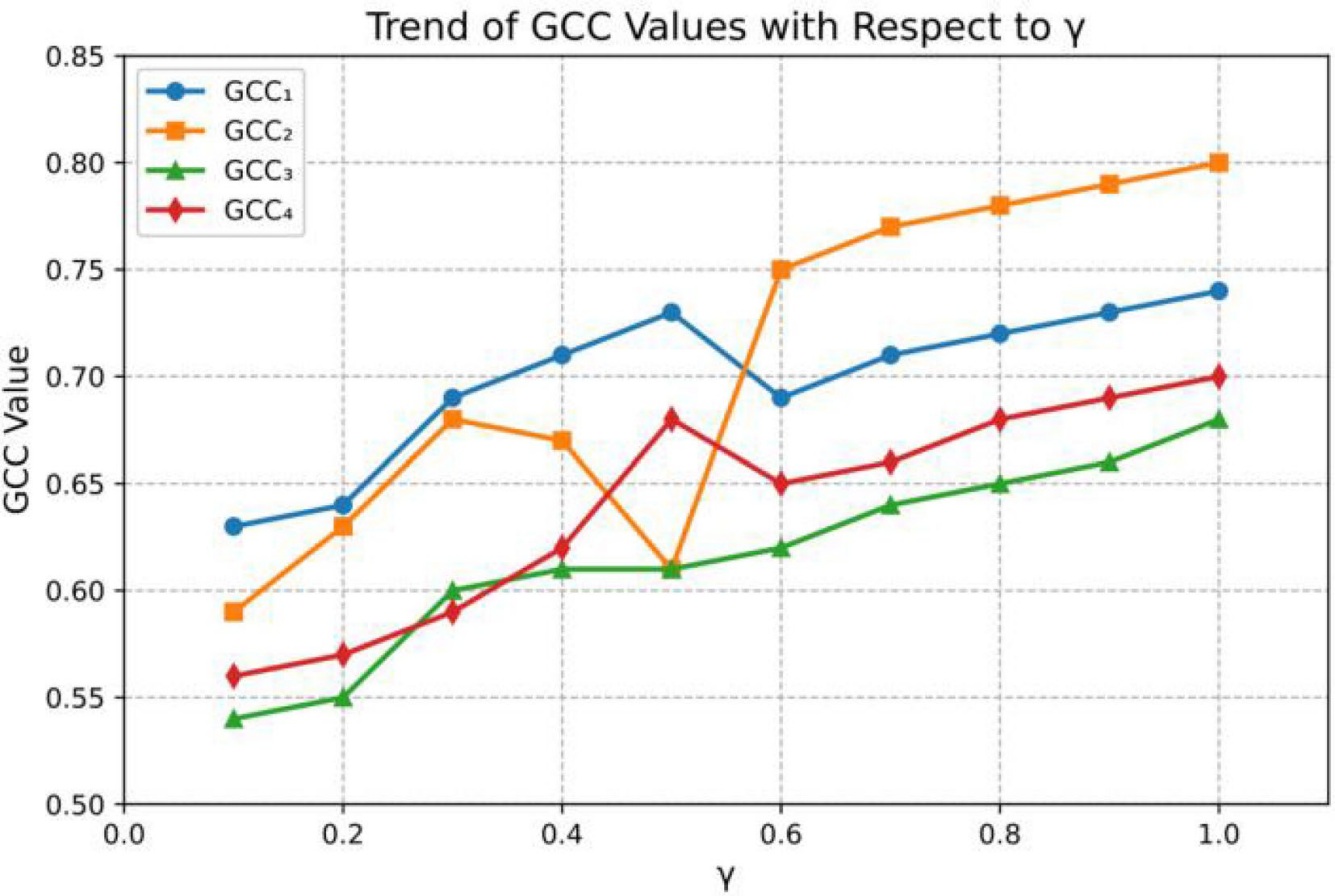
Effect of parameter $\gamma$ changes on the results under $q=3$.

This section examines how variations in key parameters influence the evaluation outcomes. Effect of changes of the parameter $\gamma$ on the results under $q=3$. The weights of attributes and $GCC_i (i=1,2,3,4)$ are obtained under different parameters, and listed in Table 47 and Fig. 2:

The parameter $\gamma$ directly determines the optimization direction by weighting the trade-off between positive class divergence (*P*(*w*)) and negative class divergence (*N*(*w*)). For small $\gamma$ values ($\gamma \le 0.4$), the objective function prioritizes minimizing *N*(*w*), leading the model to favor weight allocations that enhance similarity among negative class samples. Consequently, $\Gamma _1$ dominates due to its stability in negative class performance (evidenced by smaller $|WGNBP_{\Gamma ^-}(\Gamma _1) - WGNBP_{\Gamma ^-}(\Gamma _j)|$ values). As $\gamma$ increases ($\gamma \ge 0.6$), the focus shifts to maximizing *P*(*w*), where $\Gamma _2$ gains superiority by exhibiting stronger positive class discrimination (higher $WGNBP_{\Gamma ^+}(\Gamma _2)$ values). The transitional state at $\gamma = 0.5$, where $\Gamma _4$ temporarily surpasses $\Gamma _2$, reflects the shifting balance between these competing objectives.

The impact of $\gamma$ manifests nonlinearly through complex interactions in the *WGNBP* function’s components (e.g., modulus and inner product terms). For instance, $\Gamma _2$’s abrupt GCC improvement at $\gamma \ge 0.6$ stems from its coefficients ($M_w(\Gamma _2)$ and $IP_w(\Gamma _2, \Gamma ^+)$) exhibiting disproportionately large marginal contributions to *P*(*w*) as $\gamma$ grows, prompting the optimizer to aggressively increase its weights. Conversely, $\Gamma _3$’s consistently poor ranking arises from its weak coefficients (e.g., low $M_w(\Gamma _3)$) that minimally affect both *P*(*w*) and *N*(*w*). This nonlinearity explains why minor $\gamma$ adjustments can trigger significant ranking changes (e.g., $\Gamma _4$ overtaking $\Gamma _2$ at $\gamma =0.5$), highlighting the model’s sensitivity to parameter variations.

#### Comparisons with existing methods

(**Method 1**) As a fundamental information integration technique, aggregation operators provide a benchmark for evaluation. Our approach is first contrasted with linguistic q-rung orthopair arithmetic aggregation in q-ROF settings, yielding: $\Gamma _1=(S_{4.61}, S_{1.41}), \Gamma _2=(S_{4.72}, S_{1.28}), \Gamma _3=(S_{4.49}, S_{1.60}), \Gamma _4=(S_{4.50}, S_{1.62}).$

Scoring values demonstrate comparable outcomes: $SF\left( \Gamma _1\right) =102.84, SF\left( \Gamma _2\right) =95.11, SF\left( \Gamma _3\right) =86.27, SF\left( \Gamma _4\right) =87.10$ with identical preference ordering $\Gamma _{1}\succ \Gamma _{2}\succ \Gamma _{4}\succ \Gamma _{3}$ to our method.

(**Method 2**) Further validation is performed against linguistic q-rung orthopair geometric aggregation, producing: $\Gamma _1=(S_{4.58}, S_{1.43}), \Gamma _2=(S_{4.71}, S_{1.32}), \Gamma _3=(S_{4.48}, S_{1.61}), \Gamma _4=(S_{4.50}, S_{1.65}).$

The resultant scores: $SF\left( \Gamma _1\right) =102.29, SF\left( \Gamma _2\right) =93.06, SF\left( \Gamma _3\right) =85.56, SF\left( \Gamma _4\right) =86.66$ maintain the consistent ranking pattern $\Gamma _{1}\succ \Gamma _{2}\succ \Gamma _{4}\succ \Gamma _{3}$.

**Remarks** From the above comparisons, both the aggregation-based decision approach and our proposed method yield the same ranking order. However, while aggregation operators possess certain advantages, they exhibit significant limitations in specific scenarios that may lead to invalid or counterintuitive results.

If we modify the evaluation information for the first attribute by the first expert, transforming a decision value to (1, 0) with a LSF, the aggregation-based approach whenever is arithmetic or geometric operators will always select $\Gamma _1$ as the optimal alternative. More generally, whenever any expert assigns (1, 0) (after being transformed by a LSF) to any attribute evaluation of alternative $\Gamma _i$, the method will necessarily favor $\Gamma _i$, which is clearly unreasonable.

When the non-membership degree of any evaluation becomes zero, the aggregation results remain unchanged regardless of how other non-membership degrees are modified. For instance, transforming a decision value to $(*, 0)$ with a linguistic scale function, fixes the aggregated result for any alternative $\Gamma _i$ at $(*, 0)$, irrespective of other non-membership degree adjustments. This behavior is clearly undesirable.

(**Method 3**) Compared with TOPSIS Method

The closeness indexes obtained through the TOPSIS approach are:

$$\begin{aligned} RC(\Gamma _i)= \frac{D \left( \Gamma _i, \Gamma ^- \right) }{D \left( \Gamma _i, \Gamma ^-\right) + D \left( \Gamma _i, \Gamma ^+ \right) }, \end{aligned}$$

where,

$$\begin{aligned} D\left( \Gamma _i, \Gamma _j^+ \right)&= \frac{1}{2} \sum _{j=1}^n w_j \left( \left| \left( f(s_{\alpha _{ij}}) \right) ^3 - \left( f(s_{\alpha _j})^+ \right) ^3 \right| + \left| \left( f(s_{\beta _{ij}}) \right) ^3 - \left( f(s_{\beta _j})^+ \right) ^3 \right| \right) \\ D\left( \Gamma _i, \Gamma _j^- \right)&= \frac{1}{2} \sum _{j=1}^n w_j \left( \left| \left( f(s_{\alpha _{ij}}) \right) ^3 - \left( f(s_{\alpha _j})^- \right) ^3 \right| + \left| \left( f(s_{\beta _{ij}}) \right) ^3 - \left( f(s_{\beta _j})^- \right) ^3 \right| \right) . \end{aligned}$$

by Eq.(26), the closeness indexes are obtained and listed as follows:

$$\begin{aligned} RC(\Gamma _1)=0.81, RC(\Gamma _2)=0.59, RC(\Gamma _3)=0.18, RC(\Gamma _4)=0.26. \end{aligned}$$

Therefore, the order of alternatives is $\Gamma _{1}\succ \Gamma _{2}\succ \Gamma _{4}\succ \Gamma _{3}$.

(**Method 4**) Building upon Darko and Liang’s^48^ q-rung orthopair fuzzy EDAS framework, we implement this group decision-making technique to solve our specific problem scenario. The appraisal score is $AS_1=0.57, AS_2=0.54, AS_3=0.44, AS_4=0.52$, so the ranking result is $\Gamma _{1}\succ \Gamma _{2}\succ \Gamma _{4}\succ \Gamma _{3}$.

(**Method 5**) Building upon Krishankumar et al.’s q-ROF extension of the COPRAS group decision method^47^, this work employs the linguistic q-ROF-COPRAS framework to address our problem based q-ROF-COPRAS idea. For the specific implementation process, please refer to the literature^47^ and^54^:

Applying this linguistic q-ROF-COPRAS algorithm yields the utility degrees (UD) for all alternatives:

$$\begin{aligned} \delta _1 = 1, \delta _2 = 0.92, \delta _3 = 0.84, \delta _4 = 0.85. \end{aligned}$$

Therefore, the ranking result is $\Gamma _{1}\succ \Gamma _{2}\succ \Gamma _{4}\succ \Gamma _{3}$.

**Fig. 3:**
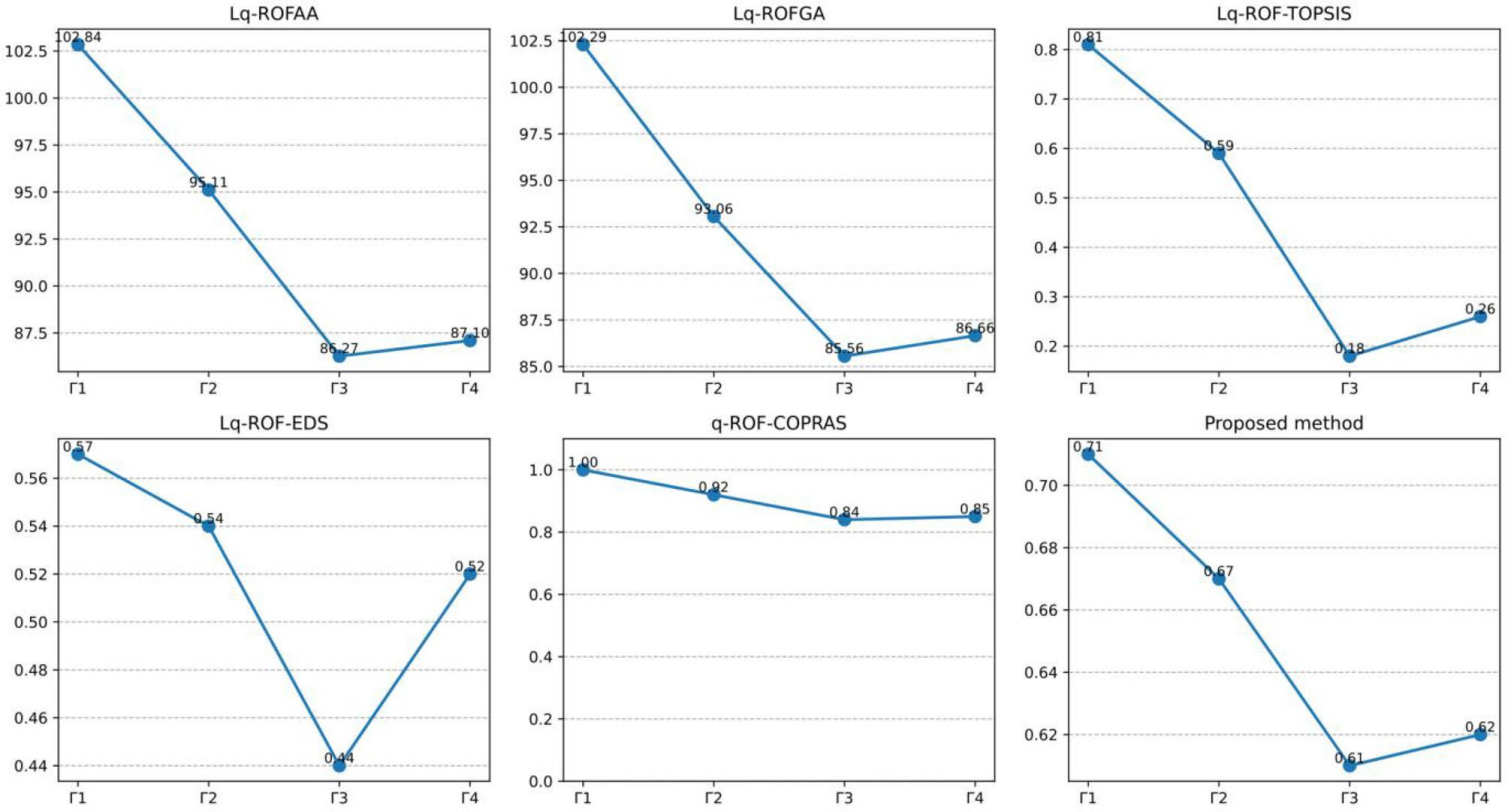
The ranks of alternatives obtained by different methods under $q=3$ and $\gamma =0.4$.

Based on the mentioned comparison and Fig. 3, the advantages of the proposed Lq-ROF Bidirectional Projection methodology based on LSFs are discussed as follows:

1. Resolution of inconsistencies via normalized bidirectional projection: Prior approaches often suffer from inconsistencies in ranking alternatives. This study proposes a novel normalized bidirectional projection measure, rigorously validated across various linguistic scale functions. This measure not only resolves these inconsistencies but also guarantees intuitive rankings and satisfies the essential normalization condition for reliable comparison.
2. Enhanced differentiation with Lq-ROF Knowledge Entropy based on LSF: Effectively differentiating nuanced linguistic assessments and dynamically determining expert credibility is critical. To address this, we develop a Lq-ROF knowledge entropy measure founded on solid axiomatic properties. This entropy measure enables precise discrimination between linguistic evaluations and provides a principled mechanism for the dynamic determination of expert weights based on the information content of their assessments.
3. Objective weight determination via integrated optimization: Rather than relying on subjective assignment, this research establishes a systematic framework for deriving objective weights. We construct a non-linear programming model to determine attribute weights, leveraging the proposed normalized bidirectional projection measure to capture inter-alternative relationships. Simultaneously, expert weights are determined based on the generalized knowledge measures, ensuring weights reflect the quality and discriminative power of the information provided.
4. Empirical validation through practical application: The feasibility and practical utility of the proposed hybrid decision-making approach, integrating the normalized bidirectional projection and generalized knowledge measures, are robustly demonstrated. A comprehensive case study assessing the selection of green contractors for shale gas development is implemented. This real-world application validates the methodology’s effectiveness in handling complex linguistic evaluations under linguistic q-ROF uncertainty.

## Conclusions

This study establishes a comprehensive Lq-ROF hybrid MCGDM framework. The principal contributions of this research are summarized as follows: (1) the introduction of a normalized bidirectional projection measure that effectively resolves ranking inconsistencies in linguistic environments while ensuring intuitive outcomes and adherence to normalization requirements; (2) the development of a theoretically-grounded q-ROF knowledge entropy measure with axiomatic foundations, enabling precise differentiation of linguistic assessments and dynamic determination of expert weights; (3) the formulation of an integrated non-linear programming model that systematically derives attribute weights through bidirectional projection while determining expert weights via generalized knowledge measures; and (4) empirical validation of the proposed integrated approach through a case study on green contractor selection for shale gas development, demonstrating its practical applicability and effectiveness.

Several limitations warrant acknowledgment and provide directions for future research: (1) the absence of an explicit consensus-building mechanism among experts during the decision process; (2) insufficient consideration of correlations within fused expert information; and (3) limited integration of comprehensive sustainability indicators within the evaluation criteria.

To address these constraints and advance the field, future research will focus on developing novel decision models in the following directions: (1) integrating behavioral decision theory with various decision methodologies to construct complex decision models under Lq-ROF environments; (2) conducting in-depth investigation of Lq-ROF preference relations to establish more robust decision structures; and (3) enhancing uncertain information representation by combining Z-numbers with Lq-ROFS to account for the reliability of expert assessments. These extensions will be applied to domains including sustainable transportation evaluation, cold-chain logistics assessment, and low-carbon technology investment.

## Data Availability

The data used in this study is included in the article. If you have further inquiries, please contact corresponding author.
